# Going Deeper: Metagenome of a Hadopelagic Microbial Community

**DOI:** 10.1371/journal.pone.0020388

**Published:** 2011-05-24

**Authors:** Emiley A. Eloe, Douglas W. Fadrosh, Mark Novotny, Lisa Zeigler Allen, Maria Kim, Mary-Jane Lombardo, Joyclyn Yee-Greenbaum, Shibu Yooseph, Eric E. Allen, Roger Lasken, Shannon J. Williamson, Douglas H. Bartlett

**Affiliations:** 1 Marine Biology Research Division, Scripps Institution of Oceanography, University of California San Diego, La Jolla, California, United States of America; 2 Microbial and Environmental Genomics, J. Craig Venter Institute, La Jolla, California, United States of America; 3 J. Craig Venter Institute, Rockville, Maryland, United States of America; 4 Division of Biological Sciences, University of California San Diego, La Jolla, California, United States of America; Universidad Miguel Hernandez, Spain

## Abstract

The paucity of sequence data from pelagic deep-ocean microbial assemblages has severely restricted molecular exploration of the largest biome on Earth. In this study, an analysis is presented of a large-scale 454-pyrosequencing metagenomic dataset from a hadopelagic environment from 6,000 m depth within the Puerto Rico Trench (PRT). A total of 145 Mbp of assembled sequence data was generated and compared to two pelagic deep ocean metagenomes and two representative surface seawater datasets from the Sargasso Sea. In a number of instances, all three deep metagenomes displayed similar trends, but were most magnified in the PRT, including enrichment in functions for two-component signal transduction mechanisms and transcriptional regulation. Overrepresented transporters in the PRT metagenome included outer membrane porins, diverse cation transporters, and di- and tri-carboxylate transporters that matched well with the prevailing catabolic processes such as butanoate, glyoxylate and dicarboxylate metabolism. A surprisingly high abundance of sulfatases for the degradation of sulfated polysaccharides were also present in the PRT. The most dramatic adaptational feature of the PRT microbes appears to be heavy metal resistance, as reflected in the large numbers of transporters present for their removal. As a complement to the metagenome approach, single-cell genomic techniques were utilized to generate partial whole-genome sequence data from four uncultivated cells from members of the dominant phyla within the PRT, Alphaproteobacteria, Gammaproteobacteria, Bacteroidetes and Planctomycetes. The single-cell sequence data provided genomic context for many of the highly abundant functional attributes identified from the PRT metagenome, as well as recruiting heavily the PRT metagenomic sequence data compared to 172 available reference marine genomes. Through these multifaceted sequence approaches, new insights have been provided into the unique functional attributes present in microbes residing in a deeper layer of the ocean far removed from the more productive sun-drenched zones above.

## Introduction

Although at one time deep oceanic environments were considered to be devoid of life, it is now well appreciated that such settings are part of the largest fraction of the biosphere, harboring the greatest numbers and diversity of aquatic microorganisms [1]. Yet despite their significance, deep ocean environments remain poorly sampled. One reflection of this is that the Global Ocean Sampling (GOS) Expedition alone has surveyed the metagenomes of 52 surface water locations [2], [3], [4], but only two pelagic deep-seawater metagenome studies have been performed to date [5], [6], [7].

Pelagic deep ocean environments are distinguished from their shallow-water counterparts in a number of fundamental physical characteristics, including the absence of sunlight, low temperature, and increased pressure with depth. Additionally, the chemical constituents of the deep ocean consist of high inorganic nutrient concentrations, such as nitrate and phosphate, and refractory dissolved organic material. The microbial biomass is largely supported by organic carbon availability, which is mainly distributed as either aggregated or dissolved sinking material exported to depth via the biological pump from the productive surface waters [8]. The microbial loop, which is well documented to exert a major influence on a variety of biogeochemical cycles in surface waters, is largely unknown in the dark ocean [9], [10].

Current information on the genomic attributes of deep-sea microorganisms from non-reducing environments has come mostly from two sources. The first is the genome sequences obtained from piezophilic (‘high-pressure adapted’) bacterial species [11], [12], [13]. Whole-genome sequence data has indicated thus far an improved capacity for complex organic polymer utilization, large numbers of transposable elements, a high ratio of rRNA operon copies per genome and larger-than-average intergenic regions [12]. The cultivated deep ocean ‘bathytypes’ have an opportunistic (r-strategy) lifestyle, allowing rapid response to environmental changes and a greater level of gene regulation [12]. While these confirmed piezophilic isolates are restricted to only a narrow phylogenetic grouping, the lifestyle strategies observed could reflect similar adaptive mechanisms across a wide range of phylogenetic types. It has recently been suggested that deep ocean microbial communities harbor functional properties indicative of ‘copiotrophs,’ separate from the streamlined high recruiting genomes found to dominate in oligotrophic surface seawater [3].

The second source of information on the genomic characteristics of deep-sea microorganisms consists of metagenomic analyses from two bathypelagic environments [5], [6]. Metagenomic approaches provide invaluable insights into the metabolic repertoire and putative functional profile of a microbial assemblage. The two bathypelagic metagenomic datasets include a 4,000 m whole-genome shotgun dataset from the Hawaii oceanographic time-series (HOT) station ALOHA (HOT4000) in the North Pacific Subtropical Gyre [6] and a 3,000 m fosmid library from the Ionian Station Km3 (DeepMed) in the Mediterranean Sea [5]. Evidence for expanded genomic repertoires in these two metagenomic datasets further supports the hypothesis that deep ocean microbes maintain an opportunistic lifestyle [5], [6]. Additionally, multiple lines of evidence suggest differential evolutionary constraints, particularly relaxed purifying (negative) selection, act upon deep-water communities compared to photic-zone counterparts [6], [7], [14]. However, these two published deep ocean metagenomes differ significantly in their particular physiochemical properties, notably in the temperature (∼1.5°C and 13.9°C for the deep station ALOHA and the Km3 site, respectively), and oceanic regimes (open-ocean gyre versus an almost landlocked basin) the microbial assemblages experience. It is therefore important to obtain additional sequence data from microbial communities residing in diverse deep ocean pelagic environments to expand the coverage and further delineate community genomic components.

The Puerto Rico Trench (PRT) is the only hadal zone (depth in excess of 6,000 m) in the northwestern Atlantic Ocean and hosts an oligotrophic water column despite the proximity to the island-arc and periodic terrigenous inputs from the adjacent continental shelf [15]. The hydrographic characteristics of the PRT include high silicate and oxygen concentrations indicative of modified Antarctic Bottom Water (AABW) from the South Atlantic Ocean [16], [17]. Our recent investigation of the PRT particle-associated and free-living microbial assemblages using small-subunit ribosomal gene libraries indicated a diverse composition of bacterial, archaeal, and eukaryal phylotypes [16].

In this study, we provide an analysis of a large-scale 454-pyrosequencing generated metagenomic dataset from the microbial community residing at 6,000 m depth within the Puerto Rico Trench (PRT). The PRT metagenome was compared against many available marine metagenomes, with an exhaustive quantitative comparison against the two bathypelagic datasets (HOT4000 and DeepMed) and two representative surface seawater datasets from the Sargasso Sea (GS00c and GS00d) [2] selected based on geographic proximity to the PRT. From these detailed quantitative comparisons, we identified unique functional attributes in the three deep-ocean microbial communities compared to the surface seawater communities. Additionally, we employed single-cell genomic techniques [18], [19] to generate genomic sequence data from four uncultivated cells from the hadal sample. The results indicate that the PRT hadopelagic microbial community has high metabolic and functional versatility reflective of adaptive mechanisms to the extreme deep ocean environment.

## Materials and Methods

### Sample collection, DNA extraction, and 454 pyrosequencing

210 l of hadal seawater was collected from 6,000 m (19.667°N, 65.966°W; bottom depth ∼8,300 m) aboard the R/V Atlantic Explorer (Bermuda Atlantic Time Series; BATS) and filtered serially through a 142 mm 3 µm TSTP (Millipore) pre-filter and a 142 mm 0.22 µm PES filter (Millipore). Hydrographic characteristics are presented in Table S1. DNA was extracted from the 0.22 µm–3 µm filter fraction as described previously [16], and was modified as in Andrews-Pfannkoch *et. al.* [20] to generate sufficient quantities of DNA for a half plate 454 Titanium pyrosequencing run (see Supplementary Methods S1).

### Assembly, functional annotation, and genome size estimation

454-generated sequencing reads were assembled using the *de novo* Newbler assembler with default parameters. Unassembled singleton reads were screened with the 454 Replicate filter (available at http://microbiomes.msu.edu/replicates/) [21] and artificial replicate sequences at a 90% sequence identity threshold were discarded (see Supplementary Methods S1). Open reading frames (orfs) greater than 90 nucleotides were called for the assembled contigs and nonredundant singleton reads using Metagene [22].

Small subunit (SSU) and large subunit (LSU) rRNAs were identified and taxonomically classified using blastn with an E-value cutoff of e^−30^ against the Silva reference database [23]. tRNAs and functional RNAs were identified using tRNAscan-SE1.23 [24] and by querying the Rfam database for non-coding and other structural RNA families [25], respectively. The taxonomic affiliations of predicted protein sequences were determined using the Automated Phylogenetic Inference System (APIS) [26], with additional functional classifications determined using the STRING v8.3 database for orthologous gene clusters (OGs) [27], [28], KEGG orthologs [29], transporter classifications (TC IDs) for membrane transport proteins [30], and Pfams [31] (see Supplementary Methods S1 for details). Estimated genome size (EGS) calculations were carried out for all metagenomes based on the method described by Raes *et. al.* [32].

### Metagenome comparisons

The unassembled nonredundant singleton reads were searched against a multitude of available marine metagenomes (Supplementary Materials and Fig. S1) using blastn with an E-value cutoff of e^−5^ to assess the similarity of the PRT sequences across various metagenomic datasets.

To quantitatively compare the gene stoichiometries in the PRT, DeepMed, HOT4000, GS00c, and GS00d datasets, a nonredundant protein dataset was generated using the blastclust algorithm as described by Konstantinidis *et. al.* [6] to reduce the effect of uneven species abundances. In order to directly compare the datasets, the DeepMed, HOT4000, GS00c, and GS00d sequencing reads were annotated and clustered to generate nonredundant protein datasets, then functionally classified as described above. Absolute abundances for the particular KOs, Pfams, and OGs were analyzed using the program ShotgunFunctionalizeR to test whether the normalized relative frequency of a particular gene family was statistically different from one metagenome compared to the other [33] (see Supplemental Methods S1).

For bacterial and archaeal OGs, the statistical techniques available within the program STAMP were utilized to assess the functional profiles annotated in the PRT metagenome compared to the surface metagenomes (GS00c and GS00d) taking into account effect size and the difference between proportions [34]. The statistical hypothesis test implemented was Fisher's exact test using the Newcombe-Wilson method for calculating confidence intervals (CIs) at the 95% nominal coverage and a Bonferroni multiple test correction. Results from these statistical methods were compared to the results obtained using the methods implemented in ShotgunFunctionalizeR and were found to be congruent in assessing the major differences in functional profiles between the PRT and surface seawater metagenomes.

### Fluorescence-activated cell sorting (FACS), whole-genome amplification, and screening of phylogenetically novel single cells

Hadal seawater collected as described previously was returned to *in situ* temperature and pressure conditions upon CTD recovery using stainless steel pressure vessels [35]. Seawater samples were maintained in 15 ml polyethylene transfer pipet bulbs (Samco) and heat-sealed with a handheld heat-sealing clamp (Nalgene) until further processing at the JCVI. High-throughput single-cell sorting was performed using a FACS-Aria II flow cytometer (BD Biosciences) equipped with a modified cooling chamber to maintain the sample at 4°C. Seawater samples were decompressed, stained for 15 min on ice with SYBR-Green I (Invitrogen), and loaded into the sample chamber with minimal exposure to fluorescent lighting. Individual cells were sorted into single wells of 384-well plates containing 4 µl TE (Tris-EDTA, pH 8.0) buffer. After sorting, plates were placed immediately at −80°C until further processing.

Cell lysis was performed using an alkaline lysis solution (645 mM KOH, 265 mM DTT, 2.65 mM EDTA pH 8.0) for 10 min on ice followed by neutralization (1290 mM Tris-HCl, pH 4.5). Handling of lysis and neutralization reagents was performed using an automated epMotion pipetting system (Eppendorf). Multiple Displacement Amplification (MDA) [36], [37] was carried out according to the manufacturer's instructions (Illustra GenomiPhi HY kit; GE Healthcare) except that reactions were incubated at 30°C for 16 h, then heat inactivated at 65°C for 3 min in a total volume of 25 µL. MDA reactions were diluted 20-fold with Tris-EDTA buffer and used as template for bacterial and archaeal 16S rRNA screening [Bacterial 16S-specific primers: 27F (5′-AGAGTTTGATYMTGGCTCAG-3′) and 1492R (5′-TACGGYTACCTTGTTACGACT-3′) and Archaeal 16S-specific primers: Arch21F (5′-TTCCGGTTGATCCYGCCGGA-3′) and Arch958R (5′-YCCGGCGGTGAMTCCAATT-3′)]. 16S rRNA screening was performed in a PCR workstation with high efficiency particulate filtered air supply. 3 µl of 1∶20 diluted template DNA was added to 17 µl Platinum Taq Supermix (Invitrogen), and PCRs were carried out on a BioRad DNA Engine thermocycler using an initial 2 min denaturation at 94°C, followed by 35 cycles of 1 min denaturation at 94°C, 30 sec primer annealing at 55°C (27F/1492R) or 50°C (Arch 21F/Arch958R), and 1 min 30 sec elongation at 72°C, with a final 10 min extension step at 72°C. Positive 16S rRNA PCR reactions were sequenced using Sanger automated cycle sequencing at the Joint Technology Center (JTC) of JCVI (Rockville, MD) as previously described [4].

### Pyrosequencing of MDA reactions and assembly

A second round of amplification was performed on MDA reactions from four phylogenetically unique single cells. Briefly, 20 ng of the original MDA DNA was used as template in a second MDA reaction using heat denaturation and cycled at 30°C for 4 hrs. Reactions were extracted using phenol-chloroform, precipitated with ethanol, and diluted to ∼50 ng/µl. 20 µg total product was used for paired-end 3 kb library construction and sequencing using the Genome Sequencer FLX System (454 Life Sciences) at the JTC. Sequence reads were screened for contamination, artificial overrepresentation and chimera formation as described in the Supplemental Material. High quality reads were assembled using Newbler and processed through the JCVI metagenomic pipeline [38].

### Fragment recruitment to marine genomes and Puerto Rico Trench single-cell genomes

The program FR-HIT [Niu and Li, unpublished; available at http://weizhong-lab.ucsd.edu/public/?q=softwares/fr-hit] was used to assess the relative number of recruited metagenomic reads from the Sargasso Sea and PRT to a reference genome database consisting of 170 sequenced genomes from the Marine Microbial Genome Sequencing Project (MMGSP; https://moore.jcvi.org/moore/), the genomes *Nitrosopumilus maritimus* SCM1 [39], Candidatus *Pelagibacter ubique* HTCC1062 [40], and the four partial genomes from the PRT single cells. The default parameters were used for FR-HIT, with a sequence identity threshold of 80%, and the output was parsed to tally the number of recruited hits to an individual genome. The unassembled reads were used after the 454-redundancy filter as described above for the PRT metagenome recruitment.

### Nucleotide sequence accession numbers

The PRT 454 metagenome has been deposited in the GenBank Sequence Read Archive under accession number SRA029331. The single-cell genomic datasets are similarly under accession numbers as follows, Rhodospirillales bacterium JCVI-SC AAA001, SRA029317; Oceanospirillales bacterium JCVI-SC AAA002, SRA029318; Flavobacteriales bacterium JCVI-SC AAA003, SRA029319; and Planctomycetes bacterium JCVI-SC AAA004, SRA029320.

## Results and Discussion

### Characteristics of a hadopelagic deep ocean metagenome

A detailed analysis of the microbial metabolic potential within the Puerto Rico Trench is presented, providing the first view of the genetic repertoire of a hadal microbial assemblage. A total of ∼145 Mbp of unique sequence data was generated with the majority consisting of unassembled singleton reads (Table 1). The average G+C content of the PRT metagenome (52.2%) was similar to that of the HOT4000 (52.1%) and DeepMed (50.1%) datasets, and distinct from the generally lower average content of surface seawater (∼36%) [6]. The estimated genome size (EGS) for the PRT was approximately 3.57 Mbp (3.07 Mbp with the exclusion of eukaryote-like sequences) (Table 2). This is in contrast to 1.75–1.85 Mbp (1.51–1.60 Mbp excluding eukaryotic sequences) for surface seawater (GS00c and GS00d). The calculated EGS for the PRT metagenome lends further support to the hypothesis that deep ocean microbial assemblages harbor, on average, larger genome sizes than their surface seawater counterparts. This could reflect the need for additional genes to cope with reduced and altered nutrients, which are more chemically diverse and biologically recalcitrant [6]. Curiously, the HOT4000 metagenome has a slightly lower EGS (2.52 Mbp excluding eukaryote-like sequences) compared to the PRT and the DeepMed metagenome (both estimated to be 3.07 Mbp excluding eukaryote-like sequences), which might be indicative of different selective pressures acting on this deep open-ocean gyre microbial assemblage.

**Table 1:** General features of the metagenomic dataset from the Puerto Rico Trench.

Feature	
Total Unique sequence (Mbp)	145.4
Total # Non-redundant contigs	25,776
Total # Non-redundant singleton reads	331,384
Largest contig (bp)	16,963
Average contig size (bp)	596
Total # SSU rRNA	496
Bacteria	463
Archaea	23
Eukarya	10
Total # LSU rRNA	1,216
Bacteria	1,196
Archaea	2
Eukarya	18
Total # tRNAs	1,884
Average G+C content (%)	52.2

**Table 2:** Functional annotations for nonredundant proteins from the PRT metagenome and comparison metagenomes.

	Sample				
Characteristic	PRT	HOT4000	DeepMed	GS00c	GS00d
Total proteins annotated	379,908	111,746	12,635	599,097	541,789
Unique protein clusters	351,799	103,569	11,304	345,380	340,046
Total matches against Pfam	146,797	87,332	7,515	234,669	226,740
Clusters with Pfam matches (PfamA)	127,721 (36.3%)	60,201 (58.1%)	5,759 (50.9%)	172,735 (50.0%)	168,214 (49.5%)
Clusters assignable to extended OGs	172,071 (48.9%)	73,025 (70.5%)	6,934 (61.3%)	203,709 (59.0%)	201,185 (59.2%)
Clusters assignable to KOs	140,571 (40.0%)	59,186 (57.1%)	5,882 (52.0%)	174,796 (50.6%)	171,227 (50.4%)
Cluster matches to PRT		76,002 (73.4%)	7,732 (68.4%)	199,620 (57.8%)	188,926 (55.6%)
Avg aa identity against PRT (%)		64.9	60.3	51.3	51.2
Estimated genome size (complete sample)	3.56	2.92	3.56	1.85	1.75
Estimated genome size (bacteria/archaea only)	3.07	2.52	3.07	1.60	1.51

Comparison of the unassembled nonredundant PRT reads against a multitude of available marine metagenomes using blastn suggests that the PRT metagenome is most similar to the HOT4000, then the DeepMed microbial communities (Fig. S1).

### Taxonomic composition of the hadal microbial community

**Figure 1:**
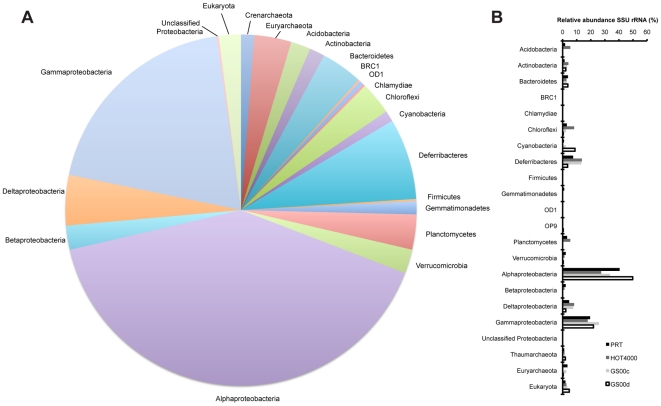
Phylogenetic distribution of partial ribosomal genes. Combined SSU and LSU rRNA genes identified from the (A) PRT metagenome, and (B) comparison of the SSU ribosomal gene distribution from the PRT, HOT4000, GS00c, and GS00d metagenomes. Phylogeny was assigned using best blastn hits to the Silva reference database (release 102).

Small-subunit ribosomal gene sequences recovered from the PRT metagenome are summarized in Figure 1. Proteobacteria, largely Alphaproteobacteria (∼40% of the total SSU and LSU ribosomal genes), dominated the PRT microbial community, which is consistent with PCR-based 16S rRNA gene sequence analyses that were conducted previously [16]. Of the large and small ribosomal subunits recovered, a strikingly small number of sequences (16), representing less than one percent of the total, were classified as belonging to the experimentally verified piezophilic Gammaproteobacterial families *Colwelliaceae* (0), *Moritellaceae* (1), *Psychromonadaceae* (6), *Shewanellaceae* (5), and *Vibrionaceae* (4). This observation suggests that the cultivated piezophiles represent only a minuscule fraction of the total autochthonous pelagic deep trench community. Members from the phyla Acidobacteria, Actinobacteria, Bacteroidetes, Chloroflexi, Deferribacteres, and Planctomycetes constituted the major remaining rRNA classifications outside of the Proteobacteria, consistent with the distribution of these groups within the HOT4000 metagenome (Fig. 1B).

Archaeal ribosomal genes were found sparingly (∼1.5% of the total), with a surprising dominance of Euryarchaeota (Marine Group II Euryarchaeota) compared to Thaumarchaeota phylotypes. These findings are in contrast to our previous archaeal 16S rRNA gene survey of the PRT, which found a dominance of Thaumarchaeota compared to the Euryarchaeota [16]. While Thaumarchaeota have generally been found to increase in abundance with depth [41], [42], [43], more recent reports suggest archaeal abundances are correlated with the particular water mass sampled [44], [45], and that Thaumarchaeota display latitudinal decline in abundances toward the equator in the North Atlantic [46]. The recovery of eukaryotic SSU and LSU ribotypes (28 sequences, 1.5% of the total), the majority of which were Basidiomycota Fungi, were similar to our prior 18S rRNA gene analyses of the PRT [16].

**Figure 2:**
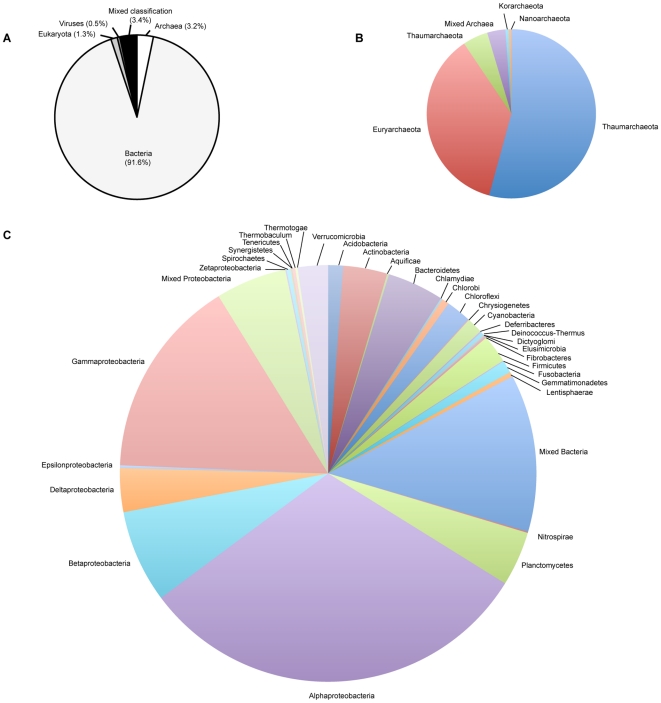
Taxonomic affiliation of protein sequences using the Automated Phylogenetic Inference System (APIS). (A) Division-level distribution. (B) Archaeal phyla. (C) Bacterial phyla with further division of Proteobacterial classes.

Taxonomic affiliation of protein sequences using the Automated Phylogenetic Inference System (APIS) [26] closely mirrored the phylogenetic distribution from the recovered ribosomal fragments. Of the proteins with phylogenomic assignments (141,722 proteins, 37.3% of the total), 91.6% were bacterial-affiliated, with the remaining 3.2% archaeal, 1.3% eukaryal, 0.5% viral-associated (3.4% could not be definitively assigned) (Fig. 2).

### Overview of the functional attributes of the PRT metagenome

**Figure 3:**
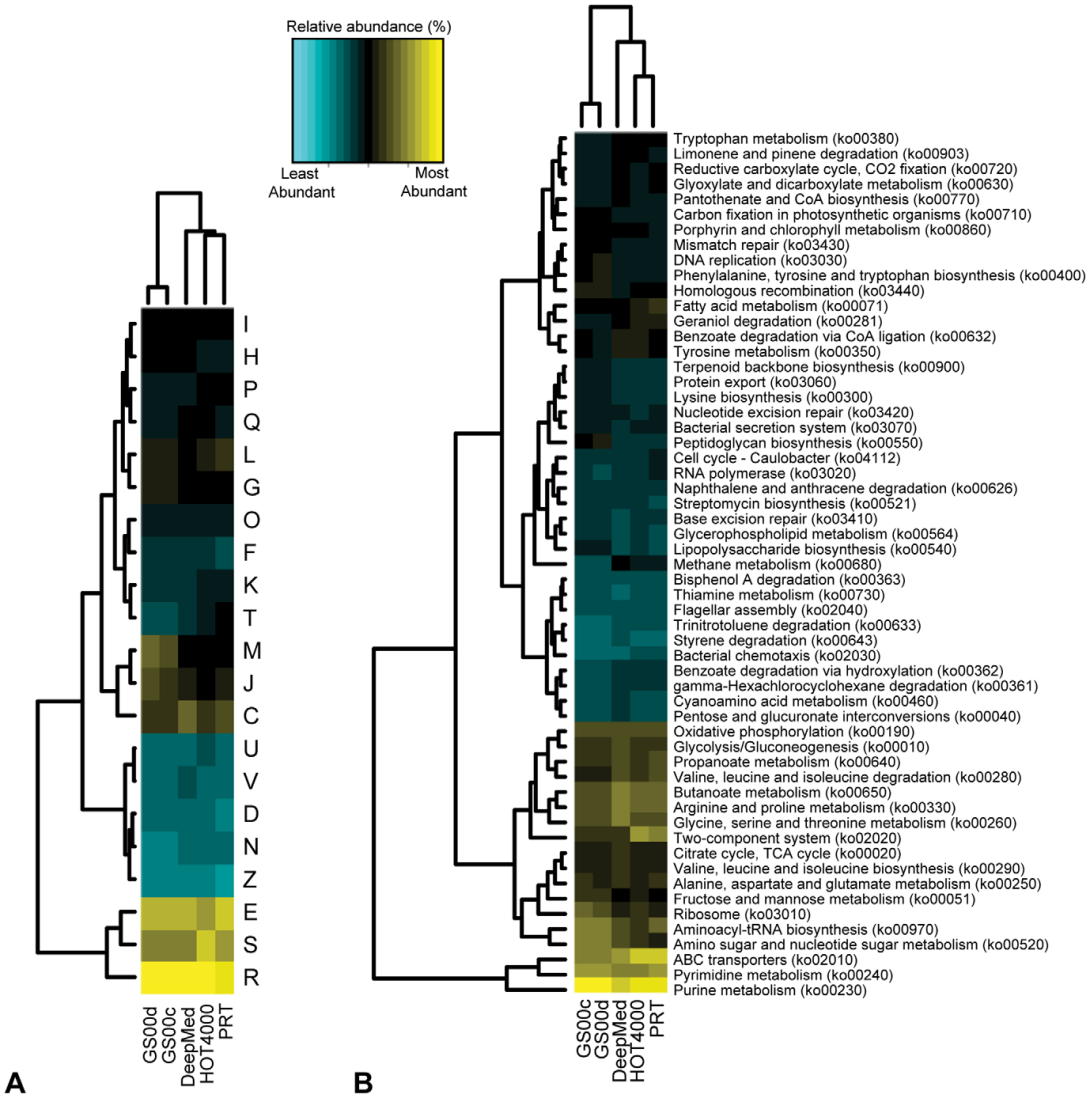
Cluster analysis of (A) COG categories and (B) KEGG pathways. Analysis was based on the relative abundances of the nonredundant protein dataset within each metagenome. Only COG categories and KEGG pathways that were represented by ≥0.2% of the total are shown. COG categories are as follows: C, energy production and conversion; D, cell division, chromosome partitioning; E, amino acid transport and metabolism; F, nucleotide transport and metabolism; G, carbohydrate transport and metabolism; H, coenzyme transport and metabolism; I, lipid transport and metabolism; J, translation and biogenesis; K, transcription; L, replication, recombination, and repair; M, cell wall/membrane/envelope; N, cell motility; O, protein turnover, chaperones; P, inorganic ion transport and metabolism; Q, secondary metabolism; R, general function prediction only; S, function unknown; T, signal transduction mechanisms; U, intracellular trafficking and secretion; V, defense mechanisms; and Z, cytoskeleton.

The relative abundances of COG categories and KEGG pathways for the nonredundant protein comparisons clustered the deep-sea apart from the surface-water datasets, with the PRT most closely related to the HOT4000 metagenome (Fig. 3). It should be noted that the construction and sequencing of these metagenomic libraries included small-insert libraries (HOT4000 and Sargasso Sea), fosmid end-sequenced libraries (DeepMed), and the PRT direct pyrosequencing library, and that certain biases associated with library construction and sequencing platform exist [47]. For example, Ghai and colleagues demonstrated cloning biases in a fosmid library compared to direct 454 pyrosequencing of the same microbial DNA collected from the deep chlorophyll maximum in the Mediterranean [48]. However, despite library construction and sequences biases, the differences observed in gene frequencies among the metagenomes being compared are consistent with the previous findings of Martín-Cuadrado *et al.* [5] and Konstantinidis *et al.* [6].

**Figure 4:**
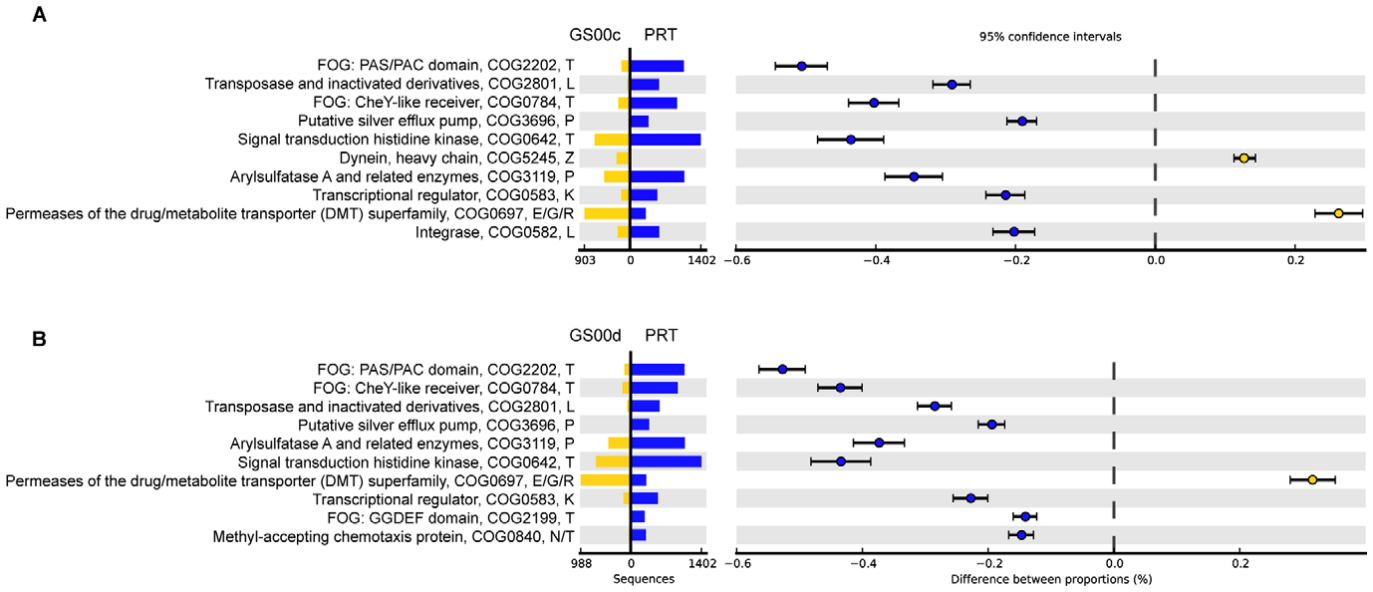
Metagenomic profile comparisons of COG families for the PRT and Sargasso Sea nonredundant proteins. Extended error bar plot for the top ten COGs, ordered according to significance, identified in the PRT compared to (A) GS00c and (B) GS00d, indicating the effect size and associated confidence intervals for each significantly different COG family. COG descriptions are listed along with the COG category letters, E, amino acid transport and metabolism; G, carbohydrate transport and metabolism; K, transcription; L, replication, recombination, and repair; N, cell motility; P, inorganic ion transport and metabolism; R, general function prediction only; T, signal transduction mechanisms; and Z, cytoskeleton.

In an effort to identify significantly different abundances of proteins and functional profiles, the nonredundant protein datasets annotated using the KEGG orthologs [29], orthologous groups (OGs) in the STRING database [27], [28], and Pfam models [31] were rigorously tested using the statistical programs ShotgunFunctionalizeR [33] and STAMP [34]. A quantitative comparison of the PRT nonredundant protein set with the two Sargasso Sea nonredundant proteins resulted in 375 and 532 orthologous groups (OGs) differentially represented in the GS00c and GS00d Sargasso Sea metagenomes, respectively (*p*<0.05 cutoff, normalized based on metagenome size and effect size; Fig. S2). As shown in Fig. 4, the metagenomic profile comparisons for the PRT and Sargasso Sea identified the most differential orthologous groups overrepresented in the PRT as falling within signal transduction mechanisms (category T), replication, recombination and repair (category L), transcription (category K) and inorganic ion transport and metabolism (category P). These results are in contrast to a pairwise comparison of the PRT and HOT4000 metagenomes, where no OGs were found to be differentially abundant (*p*<0.05 cutoff, normalized based on metagenome size and effect size).

In line with the major genomic features reported for the other two deep-ocean metagenomes, the PRT contained an over-abundance of transposable elements, a diverse complement of transporters, components for aerobic carbon monoxide (CO) oxidation, as well as oxidative carbohydrate metabolic components for butanoate, glyoxylate and dicarboxylate metabolism [5], [6], [7]. As has been observed in the HOT4000 and DeepMed metagenomes, the PRT lacked genes whose products are associated with light-driven processes, including photosynthesis, rhodopsin photoproteins, and photorepair of DNA damage. The overrepresentation of CO dehydrogenase subunits (CoxS, CoxM, CoxL), indicative of aerobic CO oxidation, further substantiates previous studies indicating that these proteins are highly represented in deep-ocean environments [5], [49]. In particular, a total of 863 CO dehydrogenase fragments, encompassing both CoxL forms I and II, were identified from the PRT. They displayed very broad phylogenetic affiliations that mirrored that of the ribosomal taxonomic distribution (mainly Proteobacteria), although only a fraction of the PRT sequences recruited to the seven CO dehydrogenase-containing DeepMed fosmids (Fig. S3) [49]. Martín-Cuadrado and colleagues [5], [49] have hypothesized that the frequencies of CO dehydrogenase genes in the bathypelagic indicate an important, albeit unclear, role for energy-generating metabolism. While the origins of CO in deep ocean environments are currently unknown, geothermal activity or the incomplete respiration of biologically labile organic matter have been proposed as potential sources [49]. Considering the PRT microbial community sampled resided more than 2,000 m above the seafloor, the most parsimonious source of CO would be the anaerobic metabolism of organic matter compared to a geothermal origin.

In general, the enzymatic components for the main autotrophic CO_2_ fixation pathways including the reductive pentose phosphate cycle (Calvin-Benson-Bassham, CBB cycle), the reductive TCA (rTCA) cycle, the 3-hydroxypropionate (3-HP) cycle, the reductive acetyl coenzyme A (acetyl-CoA) pathway (Wood–Ljungdahl pathway), the 3-hydroxypropionate/4-hydroxybutyrate (3-HP/4-HB) cycle, and the dicarboxylate/4-hydroxybutyrate cycle were represented in the PRT metagenome with comparable abundances in both the deep and surface seawater metagenomes. However, key enzymes from some of these pathways were either poorly represented or missing in the PRT. Only two low-identity matches to ATP-citrate lyase (EC 2.3.3.8), the key enzyme for the rTCA cycle, were identified, as well as sixteen hits to ribulose-bisphosphate carboxylase (EC 4.1.1.39) and four hits to phosphoribulokinase (EC 2.7.1.19), the key enzymes of the CBB cycle. Of the matches to ribulose-bisphosphate carboxylase (RubisCO), the majority of the proteins resembled the archaeal type III or type IV RubisCO-like protein (RLP) homologs, which have alternative functions for sulfur metabolism [50], [51]. This in contrast to the type I and II RubisCO homologs identified in the surface seawater nonredundant proteins, as well as from reducing environments like hydrothermal vent chimneys that are predominantly fueled by autotrophic carbon fixation via the CBB pathway [52]. Importantly, the major enzymes necessary for the 3-HP and 3-HP/4-HB pathways, 3-hydroxypropionate dehydrogenase (EC 1.1.1.298) and malonyl-CoA reductase (EC 1.2.1.75), were absent from the PRT dataset. As a result of the absence or limited abundance of sequences encoding these enzymes, it is possible that these autotrophic carbon fixation pathways play a minor role compared to heterotrophic metabolic strategies.

### Features overrepresented in the deep ocean, and in particular the hadopelagic, identified through comparative metagenomics

#### Signal transduction mechanisms (Category T)

Two-component signal transduction is a stimuli-response mechanism important for microorganisms to cope with changing environmental conditions. Positive correlations in genome size and enrichment of signal transduction functions have been reported, as well as poor representation of these functions in the dominant surface marine bacterial genomes [3], [53]. The PRT nonredundant protein set was highly enriched in signal transduction functions, particularly FOG: PAS/PAC domain proteins (COG2202), FOG: CheY-like receiver protein (COG0784), signal transduction histidine kinases (COG0642), and FOG: GGDEF, EAL, and GAF domain proteins (COG2199, COG5001, COG2200, and COG2203) (Fig. S4). The PAS/PAC domain proteins (COG2202) were particularly enriched in the PRT. PAS domain-containing proteins are located in the cytosol where they function as internal sensors of redox potential and oxygen [54]. These findings support the proposed hypothesis that deep ocean microbial assemblages possess functions to cope with resource scarcity and a high diversity of molecular substrates. While the HOT4000 metagenome was enriched in the sensory domain-containing proteins listed above compared to the Sargasso Sea, the PRT metagenome had a more prominent enrichment in all cases, which could suggest a requirement for increasing numbers of signal transduction pathways with increasing depth.

#### Transcription (Category K)

The deep ocean nonredundant proteins, and the PRT in particular, displayed an enrichment of transcriptional regulators (COG0583, transcriptional regulator; COG2207, AraC-type DNA binding domain-containing protein; COG1595, DNA-directed RNA polymerase specialized sigma subunit sigma24 homolog; COG0640, predicted transcriptional regulators; and others) (Fig. S5). The enrichment of COG1595, the specialized sigma24 homolog, or *rpoE*-like, is intriguing since this alternative RNA polymerase sigma factor plays a role in outer membrane protein synthesis and growth at low-temperature, high-pressure conditions in the piezophile *Photobacterium profundum* SS9 [55]. In general, these are trends for transcriptional regulation functionalities representative of a copiotrophic lifestyle strategy [56] found within the PRT metagenome and the two other deep ocean metagenomes. Expanded gene families for transcription and in particular, transcriptional regulation, are important features in the piezophile and piezotolerant genomes *P. profundum* SS9 and *Shewanella piezotolerans* WP3, respectively [11], [13].

#### Inorganic ion transport and metabolism (Category P)

One striking example within category P is the overrepresentation of arylsulfatase A and related enzymes (COG3119) in the deep nonredundant proteins, particularly for the PRT metagenome (Fig. S6). Sulfatases catalyze the hydrolysis of sulfate esters for the degradation of sulfated polysaccharides, and are highly abundant in the phyla Planctomycetes and Lentisphaerae [57], [58]. Notably, the piezotolerant bacterium *S. piezotolerans* WP3 contains eight putative sulfatase genes [11]. While the role of these numerous sulfatases is not clear, one hypothesis based on studies of *Rhodopirellula baltica* [59] suggests that in addition to polymer degradation, sulfatases are involved in structural remodeling during morphological differentiation. Interestingly, when the orthologous groups were further divided into phylum-level groupings based on the APIS classifications, the representation of sulfatases was found to be highly abundant within the phylum Lentisphaerae (Fig. S7).

#### Transporters

**Figure 5:**
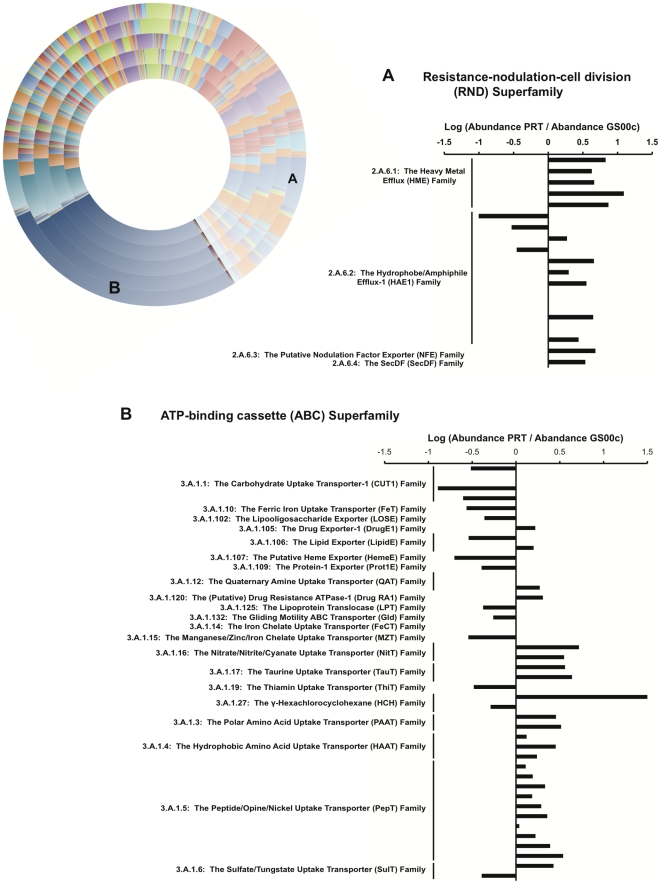
Transporter family distribution. Outer circle moving inwards: PRT, HOT4000, DeepMed, GS00d, GS00c. Log abundance profiles for (A) the Resistance-nodulation-cell division (RND) superfamily and (B) the ATP-binding cassette (ABC) superfamily are shown for the PRT compared to the GS00c. Positive values in the bar chart denote greater abundances in the PRT, while negative values are greater abundances in the GS00c for the given transporter family member. Similar results were observed for PRT and GS00d metagenome comparisons. Transporter classification details are included in the Supplementary Materials.

Transport mechanisms are one of the major cellular processes differentially influenced by hydrostatic pressure changes in transcriptomic analyses in the piezophilic bacterium *P. profundum* strain SS9 [60]. Additionally, the most pressure-regulated proteins produced by this bacterium are outer membrane porin proteins [61], [62]. Since high hydrostatic pressure acts to reduce the system volume, and consequently modifies the cellular membrane, the structural diversity, specificity, and variety of transporters would presumably be distinct in deep ocean microbial assemblages compared to surface seawater counterparts. To address this question, the nonredundant protein sets were classified using the Transporter Classification Database (TCDB) [30]. The overall diversity of transporter families identified was comparable across the metagenomes, generally with an even distribution and representation (Fig. 5). However, comparisons of the deep and surface seawater metagenomes revealed that 281 transporter classifications (TC IDs) within 116 transporter families (out of a total of 610 transporter families) were significantly different (*p*<0.05), and 155 of the 281 TC IDs were enriched in the deep-sea compared to the shallow-water datasets. These included the general secretory and outer membrane protein secreting pathways, many outer membrane proteins (most are members of the outer membrane receptor family, the outer membrane porin family, the OmpA-OmpF porin family, and the FadL outer membrane family), diverse cation transporters (sodium symporters, monovalent cation antiporters, cation diffusion facilitators, ferrous iron and magnesium transporters), including many associated with heavy metals (chromate, arsenical resistance family, resistance-nodulation-cell division, arsenite-antimonite efflux, iron lead transporters, mercuric ion permeases, P-type ATPases). Also enriched were peptide transporters, including those linked with carbon starvation, mono, di- and tri-carboxylate transporters, mechanosensitive ion channels, members of the major facilitator superfamily, tripartite ATP-independent periplasmic transporters and ATP-binding cassette superfamily transporters (Supplementary Table S2).

The largest, significantly different group of ABC transporter families overrepresented in the deep ocean metagenomes were of the Peptide/Opine/Nickel Uptake Transporter (PepT) Family (TC3.A.1.5), including non-specific oligopeptide transport, glutathione porter, and probable rhamnose and xylose porters (Fig. 5B). The deep metagenomes, and the PRT in particular, were also enriched in nitrogen uptake transporters of the families Nitrate/Nitrite/Cyanate (NitT; TC3.A.1.16), Quaternary Amine (QAT; TC3.A.1.12), Taurine (TauT; TC3.A.1.17), and the nitrate/nitrite porter (NNP; TC2.A.1.8.2). The total organic nitrogen (TON) concentration in the PRT was significantly higher compared to the overlying surface seawater (∼29 µM compared to 5.7 µM), as was the inorganic nitrate concentration [16]. The enrichment in nitrogen uptake systems is therefore congruent with the greater concentration of nitrogen and indicates the ability of the PRT microbial assemblage to utilize this nitrogen source.

All transporters classified within the Heavy Metal Efflux (HME) Family (TC2.A.6.1), within the Resistance-Nodulation-Cell Division (RND) Superfamily, were overrepresented in the PRT compared to the Sargasso Sea (Fig. 5A). This is an intriguing finding considering that the majority of heavy metal efflux systems currently characterized are found within contaminated environments [63]. The overrepresentation of heavy metal efflux pumps (COG3696) in the PRT metagenome compared to surface seawater datasets is observed in the two other deep ocean datasets, yet is more pronounced in the PRT metagenome comparisons (Fig. S6). These include efflux systems such as the CzcCBA and CusCFBA H^+^-antiport systems for Ni^2+^, Co^2+^, Zn^2+^, Cd^2+^, Cu^+^, and Ag^+^ efflux [64]. Additionally, nine families within the P-type ATPase Superfamily (TC3.A.3) were significantly overrepresented, all more abundant in the deep and almost all associated with heavy metal translocation for Cu^+^, Ag^+^, Zn^2+^, and Cd^2+^. The enrichment of both H^+^ and ATP-driven efflux systems indicates diverse mechanisms to deal with elevated concentrations of trace metals in the hadopelagic.

Dramatic changes in the elemental composition of sinking particulates with depth have been documented in the Sargasso Sea, where sinking material (such as marine snow) can become rapidly depleted in organic matter, while becoming enriched in lithogenic, authigenic minerals, and redox sensitive elements that are scavenged on particles [65], [66]. The chemical speciation of trace metals (free hydrated ions, inorganic complexes, and organic complexes) in the deep ocean is poorly understood, which has dramatic consequences for the different biogeochemical interactions of the microbial assemblage. Total and free (bioavailable) copper concentrations are highly elevated in the deep ocean relative to the surface ocean, which could necessitate the particular efflux pumps identified from the PRT metagenome [67], [68]. Although sample processing included pre-filtration through a 3 µm-pore size filter, the possibility of capturing microbial members of the particle-associated community might explain the enrichment for functions associated with heavy metal resistance, as well as other surface-associated genomic features, and the high GC content as first proposed by Martín-Cuadrado and colleagues [69].

The elemental composition of not only sinking particulates, but also neutrally-buoyant macroscopic particles might also call for a diverse array of heavy metal efflux systems in the deep. Bochdansky and colleagues found pronounced peaks of macroscopic particles (>500 um) within the deep Antarctic Bottom water (AABW) and two branches of the North Atlantic Deep Water (NADW), suggesting these macroscopic particles can act as microbial ‘hot-spots’ [70]. Similarly, Ivars-Martinez *et al.* (2008) found an enrichment of genomic features associated with heavy metal resistance in the deep *Alteromonas macleodii* ecotype compared to the surface-dwelling ecotype, as well as demonstrating that most deep *Alteromonas* isolates were more heavy metal resistant compared to their shallow-water counterparts [71].

In addition to the proposed impact of laterally-advected macroscopic particles entrained within the AABW, which flows into the PRT from the South Atlantic, the unique topography of the PRT lends itself to turbidity flows and inputs of terrigenous detritus from the nearby continental shelf [15]. These particular oceanographic considerations undoubtedly contribute to the types of functional features of this unique hadal microbial assemblage. Further work is clearly needed to investigate the association between the enrichment of heavy metal efflux systems in deep ocean microbial assemblages and the specific concentrations (and speciation) of trace metals in hadal environments.

#### Multiple Displacement Amplification (MDA) of four uncultivated single cells from the PRT

Single-cell genomics was pursed as a route to obtain further insight into the PRT community. Single cells were isolated by fluorescence activated cell sorting (FACS) and their DNA amplified using Multiple Displacement Amplification (MDA) [18], [19]. Four unique uncultivated bacterial cells were selected for sequencing (Table 3). Although genome recovery was minimal, for reasons likely related to DNA damage associated with sample handling and cell lysis for MDA, the data obtained exceeded the single-cell sequence information available in fosmid clones derived from environmental DNA (See Supplementary Methods S1, Table S3). The phylogenetic affiliations of the four single cells are detailed in Table 4 and Supplementary Figure S8.

**Table 3:** Single cell sequencing, assembly, and annotation statistics.

	Alphaproteobacterium Rhodospirillales bacterium JCVI-SC AAA001	Gammaproteobacterium Oceanospirillales bacterium JCVI-SC AAA002	Bacteroidetes Flavobacteriales bacterium JCVI-SC AAA003	Planctomycetes bacterium JCVI-SC AAA004
# Reads Input	113,421	189,378	130,439	42,704
# Bases Input	24,465,204	37,000,539	27,133,706	11,443,900
Total # Contigs	249	66	84	144
% Chimeric Reads	15.42	9.9	16.05	50.21
Total # Non- contaminant contigs	42	9	14	13
Total sequence after filters (kbp)	310	190	209	58
Largest contig (kbp)	51	71	114	24
Total # Non-contaminant orfs	276	159	182	48

**Table 4:** Comparison of single cell phylogeny to closest sequenced isolate genome.

Single cell phylogeny	Closest sequenced relative with finished genome (16S rRNA % ID)	Top BLAST hit NCBI nr (16S rRNA % ID; reference)
Gammaproteobacterium Oceanospirillales bacterium JCVI-SC AAA002	*Kangiella koreensis* DSM 16069 (90%)	EU287377, Arctic sediment (98%; [83])
Alphaproteobacterium Rhodospirillales bacterium JCVI-SC AAA001	*Magnetospirillum magneticum* AMB-1 (88%)	EU919770, Arctic ocean (92%; [84])
	*Rhodospirillum centenum* SW (86%)	
	*Rhodospirillum rubrum* ATCC 11170 (86%)	
Bacteroidetes Flavobacteriales bacterium JCVI-SC AAA003	*Flavobacterium johnsoniae* UW101 (88%)	EU919825, Arctic Ocean (96%; [84])
	*Flavobacterium psychrophilum* JIP01/86 (87%)	
Planctomycetes bacterium JCVI-SC AAA004	*Rhodopirellula baltica* SH 1 (81%)	HM799119, PRT seawater (100%; [16])

#### Alphaproteobacterium

The Rhodospirillales bacterium JCVI-SC AAA001 single cell sequence data was the most complete of the four single cells studied, consisting of 310 kbp of assembled sequence data (Table 3). A complete methionine biosynthetic pathway was identified with an initial succinylation step catalyzed by homoserine trans-succinylase (HTS – EC 2.3.1.46). The sulfur inclusion second step could proceed through direction incorporation of sulfide (sulfhydrylation) to form homocysteine either via a putative sulfhydrolase (contig00083 - Cystathionine beta-lyase/cystathionine gamma-synthase) or an intermediate associated with the cysteine synthase A (contig00025). Some organisms have active copies of the CGS (cystathionine gamma-synthase), CBL (Cystathionine beta-lyase), and HS (homocysteine synthase) and are capable of carrying out both direct incorporation and transsulfuration capacities [72]. Lastly, methylation of the homocysteine via an identified cobalamin-dependent methionine synthase completed the pathway. Additionally, a SAM (S-adenosylmethionine) riboswitch (RF00521) was identified on contig00002, which could be associated with the methionine biosynthetic genes identified on various other contigs. The riboswitch identified is a SAM-II aptamer with close identity and structure to other riboswitches found predominantly in Alphaproteobacteria [73].

A two component system for regulating transport and catabolism of phosphorous-containing compounds (PhoBR) and an ABC transporter for P_i_ compounds was identified (contig00193). The phosphorous concentrations measured in the PRT were two-orders of magnitude greater than the overlying surface seawater [16], so presumably the ability to utilize phosphorous efficiently is an important metabolic feature in the deep. There were also three components of the ABC-Fe^3+^ transport system (AfuBCA) (contig00162), a high-affinity TRAP C4-dicarboxylate transport (Dct) system utilizing an electrochemical gradient instead of ATP (contig00059), and assimilatory nitrogen metabolic components, such as nitrate and nitrite reductases in addition to an ABC-type transport system for nitrate/sulfonate/bicarbonate (contig00165). Interestingly, the Pho genes were flanked by integrase sequences, and transposase IS sequences flanked the nitrate and nitrite transporters as well, suggesting that mobile genetic elements may influence the transfer of these and other genes between members of the deep ocean microbial community.

#### Gammaproteobacterium

The genomic information recovered from the Oceanospirillales bacterium JCVI-SC AAA002 single cell consisted of 190 kbp of assembled sequence data (Table 3) and had the closest reference genome match to *Kangiella koreensis* DSM 16069, isolated from a tidal flat using dilution-to-extinction culturing in a rich marine medium [74]. A complete biotin biosynthetic cluster was identified (*bioBFHCD*, contig00007) along with multiple ribosomal proteins localized on two main contigs (contig00048 and contig00049). Interestingly, a system for copper homeostatis (*copAB* copper resistance proteins) and efflux (*cusCBA*) were identified (contig00022), providing support for the overrepresentation of H^+^ and P-type ATPase heavy metal efflux systems in the PRT metagenomic data.

#### Bacteroidetes

The Flavobacteriales bacterium JCVI-SC AAA003 single cell sequence data had the longest contig of the four single cells (114 kbp of a total 209 kbp recovered) and encoded 182 putative orfs (Table 3). A complete ribosomal operon (5S-23S-Ile.tRNA-Ala.tRNA-16S) was present on the largest assembled contig (contig00082), with two additional tRNAs identified (Arg and Val). The closest completed reference genomes are *Flavobacterium johnsoniae* UW101 (6.1 Mbp) and *Flavobacterium psychrophilum* JIP01/86, (2.86 Mbp).

A complete assimilatory nitrogen metabolic pathway was recovered (contig00082) for the conversion of nitrate to L-glutamate, with the identified enzymatic components including nitrate and nitrite reductases, glutamine synthetase (EC6.3.1.2), and glutamate synthase (EC1.4.7.1). As with the Alphaproteobacterium single cell, the identification of nitrogen uptake components gives genomic context to the functional enrichment of nitrogen uptake systems identified within the PRT metagenome and is congruent with the greater concentration of nitrogen in the hadopelagic. Also of interest were multiple putative sulfatases and sulfatase precursors, including a putative arylsulfatase with closest sequence similarity to a *Robiginitalea biformata* HTCC2501 sulfatase ([75]; NCBI locus: YP_003196467), a putative secreted sulfatase *ydeN* precursor with closest similarity to *Lentisphaerae araneosa* HTCC2155 ([58]; NCBI locus: ZP_01872651), and two truncated iduronate-2- sulfatase precursors also with closest similarity to *Lentisphaerae araneosa* HTCC2155 ([58]; NCBI locus: ZP_01873063). The representation of multiple sulfatases from the Bacteroidetes single cell and the overrepresentation of sulfatases in PRT metagenome further supports the hypothesis that there is a potentially multifaceted role for these enzymes in the hadopelagic, including polymer degradation and structural remodeling of the cell wall.

#### Planctomycetes

The recovered Planctomycetes bacterium JCVI-SC AAA004 single cell sequence data was the most fragmentary, with 48 putative proteins (32 with annotations) encoded on thirteen contigs. The Planctomycetes phylum in general is underrepresented in the sequence databanks, currently with 13 genome projects (GOLD [76], October 2010; of which only four are closed and finished) and a handful of fosmids [77], [78]. Consistent with the genome architecture and genomic repertoire of the marine Planctomycetes, the PRT Planctomycetes bacterium contains numerous hypothetical genes and a lack of apparent operon structure for essential pathways. Eleven hypothetical proteins were identified, one of which contained DUF1570 (PF07607), a family of hypothetical proteins in *Rhodopirellula baltica* SH1^T^ [57]. The sequenced Planctomycetes contain unlinked *rrn* operons, for example, the *Rhodopirellula baltica* SH1^T^ genome contains a 460 kbp region separating the 16S from the 23S-5S [57]. We were unable to assess whether the single cell rRNA operon was linked or not, since the 16S rRNA and 23S-5S rRNA genes were not located on the same contig. The flagellar biosynthesis gene *flhF* and a putative flagellar RNA polymerase sigma factor (RNA polymerase sigma factor *whiG/fliA*) were present, suggestive of a motile lifestyle.

#### High recruitment of the PRT metagenome to the PRT single cells

**Figure 6:**
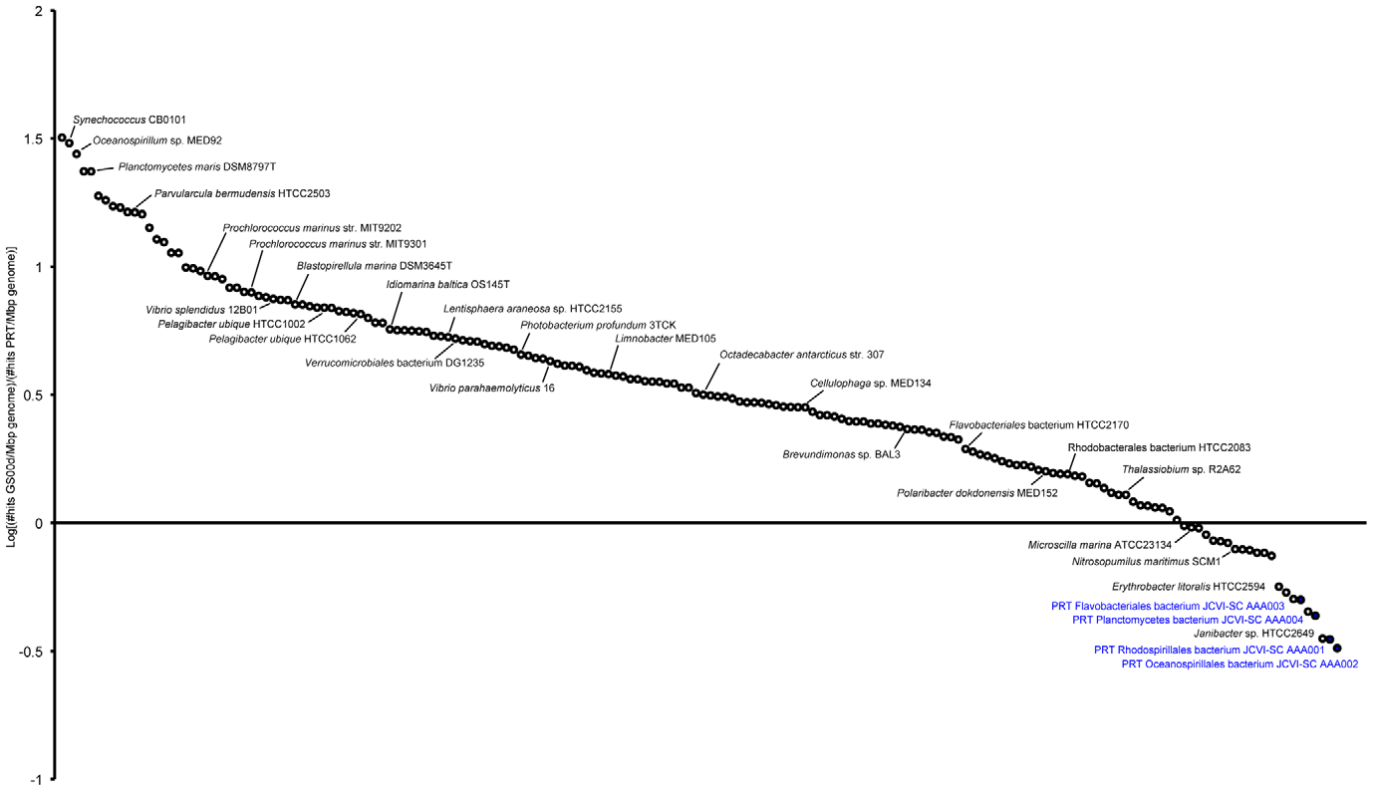
Comparative fragment recruitment for the PRT and GS00d metagenomes. Recruitment of the PRT metagenome compared to the GS00d metagenome to the Marine Microbial Genome Sequencing Project (MMGSP) genomes, *Nitrosopumilus maritimus* SCM1 [39], Candidatus *Pelagibacter ubique* HTCC1062 [40], and the four PRT single-cell genomes. Circles represent reference genomes. Reference genomes with greater normalized relative recruitment (sequence identity threshold 80%) to the PRT are below the zero line, while genomes above the zero are better recruiters for the GS00d dataset. In calculating the ratio of GS00d/PRT recruitment, the number of reads recruited from each metagenome was normalized to the size (in Mbp) of the reference genome as well as to the number of total reads in the metagenome. Similar results were observed for PRT and GS00c metagenome comparisons.

Fragment recruitment of the PRT raw metagenomic reads to the four single cells yielded extremely high recruitment compared to recruitment to 172 sequenced marine microbial genomes (Fig. 6). The majority of PRT reads which recruited to the single-cell genomes were matches to portions of the ribosomal operons, with percent identity ranging from 84.5 to 88.0%, as well as transfer-RNA sequences. The Planctomycetes bacterium JCVI-SC AAA004 was the only single cell to recruit fragments of the PRT metagenome to all contigs of the dataset, in addition to recruiting the most reads relative to the size of the genome for any of the genomes compared (45,373 hits/Mbp).

The number of reads recruited from the PRT metagenome to any given reference marine genome was low compared to the Sargasso Sea metagenome (Fig. 6). Thus, despite the poor recovery of genome sequence data from the four single cells, the high level of PRT metagenome recruitment demonstrates both the low representation of deep-ocean microbial genomes currently available and the power of single-cell genomics to complement metagenomic coverage of an environment. A similar analysis was performed for the HOT4000 dataset and demonstrated relatively high recruitment to the four PRT single cells, although not as heavily as the PRT metagenome recruitment. These data indicate the distinct composition of deep-ocean microbial genomes, which are not well represented in currently available marine microbial genome sequences.

### Summary

This study has provided the first large-scale molecular sequence dataset from a hadopelagic environment. The data demonstrate that the PRT microbial community possesses larger genomes that are enriched in signal transduction, particularly PAS domain-containing proteins that function as internal sensors of redox potential and oxygen, transcriptional regulators and alternative sigma factors like RpoE that have been shown to play a role in growth at low-temperature and high-pressure, and transposable elements. A distinctive collection of transporter mechanisms was identified, including numerous transporters associated with heavy metal resistance. An overabundance of metabolic pathways associated with aerobic carbon monoxide (CO) oxidation and oxidative carbohydrate metabolism was present in the PRT dataset, along with sulfated polysaccharide degradation, which was particularly prevalent within the PRT members of the phylum Lentisphaerae. Partial single-cell genomes from members of the PRT Alphaproteobacteria, Gammaproteobacteria, Bacteroidetes and Planctomycetes were investigated and found to highly recruit the PRT metagenome gene sequences, as well as providing further genomic context to some of the trends observed in the PRT metagenome. Future work to delineate the metabolic potential from other deep-ocean environments and single-cells, as well as cultivation approaches to obtain a more phylogenetically-diverse sets of reference piezophiles, will shed further light on the diversity, evolution and adaptations of microbial life in the dark ocean.

## Supporting Information

Figure S1 Comparison of the unassembled nonredundant PRT reads against the DeepMed [5]; a 7-depth profile from Station ALOHA (10 m, 70 m, 130 m, 200 m, 500 m, 770 m, 4,000 m) [7]; the Mediterranean deep chlorophyll maximum (DCM) [48]; 1,300 m depth sediment and 1,000 m depth water column from the Sea of Marmara [79]; black smoker chimney in the Mothra hydrothermal vent field at the Juan de Fuca Ridge [52]; the Peru Margin subseafloor [80]; and a subset of sites from the Sargasso Sea pilot study (GS00c and GS00d) [2] and the Global Ocean Survey (GS03, North American East Coast; GS04, North American East Coast; GS05, North American East Coast; GS16, Caribbean Sea; GS17, Caribbean Sea; GS18, Caribbean Sea; GS23, Eastern Tropical Pacific; GS37, Eastern Tropical Pacific; GS122a, Indian Ocean; GS123, Indian Ocean) [3], [4]. The number of top BLAST hits was normalized to the size of the comparison metagenome.
(PDF)

Figure S2 Statistical hypothesis testing implemented in the program STAMP [34] for differentially abundant orthologous groups (OGs) between the PRT metagenome and the Sargasso Sea (A) GS00c and (B) GS00d metagenomes. Results are shown for the Fisher's exact test using the Newcombe-Wilson method for calculating confidence intervals (CIs) at the 95% nominal coverage and a Bonferroni multiple test correction. Initially, 436 (PRT vs. GS00c) and 592 (PRT vs. GS00d) OGs were identified having significant differences (*p*<0.05). Subsequent filtering of these significant differences was performed taking into account effect size, with the difference between proportions set to a value of 0.5% and the ratio of proportions set to 2.0, resulting in 375 (PRT vs. GS00c) and 532 (PRT vs. GS00d) significantly different OGs represented.
(PDF)

Figure S3 Fragment recruitment coverage plots for the unassembled nonredundant PRT metagenomic reads against the seven fully sequenced fosmids from Martín-Cuadrado *et al.* [49]. (A) KM3-26-C03 (NCBI Accession number: GU058051), (B) KM3-28-H12 (GU058052), (C) KM3-29-C02 (GU058053), (D) KM3-41-E12 (GU058054), (E) KM3-45-H11 (GU058057), (F) KM3-54-A05 (GU058055), (G) KM3-60-B01 (GU058056). Fragment recruitment was carried out using blastn as described by Rusch *et al.* [4]. Fosmid gene maps and annotations are shown as in Martín-Cuadrado *et al.* [49]. Coverage (blue bars) represents sequencing depth across the given fosmid, while % Identity (black circles) represents the percent sequence identity of the recruited PRT reads.
(PDF)

Figure S4 Abundance of the functional OG category Signal Transduction (T) for deep ocean metagenomes compared to the Sargasso Sea metagenomes.
(PDF)

Figure S5 Abundance of the functional OG category Transcription (K) for deep ocean metagenomes compared to the Sargasso Sea metagenomes.
(PDF)

Figure S6 Abundance of the functional OG category Inorganic ion transport and metabolism (P) for deep ocean metagenomes compared to the Sargasso Sea metagenomes.
(PDF)

Figure S7 Relative abundance of assignable COG categories and distribution within phylum-level (and class-level for the Proteobacteria) groupings based on APIS. Only phyla contributing ≥0.2% of the total proteins classified are shown. COG categories are as follows: A, RNA processing and modification; B, chromatin structure and dynamics; C, energy production and conversion; D, cell division, chromosome partitioning; E, amino acid transport and metabolism; F, nucleotide transport and metabolism; G, carbohydrate transport and metabolism; H, coenzyme transport and metabolism; I, lipid transport and metabolism; J, translation and biogenesis; K, transcription; L, replication, recombination, and repair; M, cell wall/membrane/envelope; N, cell motility; O, protein turnover, chaperones; P, inorganic ion transport and metabolism; Q, secondary metabolism; R, general function prediction only; S, function unknown; T, signal transduction mechanisms; U, intracellular trafficking and secretion; V, defense mechanisms; and Z, cytoskeleton.
(PDF)

Figure S8 Phylogenetic trees depicting the relationship of the 16S rRNA gene sequences for the (A) Alphaproteobacterium Rhodospirillales bacterium JCVI-SC AAA001, (B) Gammaproteobacterium Oceanospirillales bacterium JCVI-SC AAA002, (C) Bacteroidetes Flavobacteriales bacterium JCVI-SC AAA003, and (D) Planctomycetes bacterium JCVI-SC AAA004. The rRNA gene sequences were aligned using the SINA Webaligner [23], uploaded into the ARB program [81] and manually checked with the ARB_EDIT4 tool. Aligned sequences were exported for bootstrap analysis using PHYLIP [82] for the neighbor joining method. Bootstrap support (1000 replicates) for nodes are indicated for values >50%. The outgroups used to calculate phylogeny were *Bacillus subtilis* 168 (AL009126) and *Escherichia coli* K-12 (U00096).
(PDF)

Table S1 Chemical and biological constituents of hadal (6,000 m) seawater. Data previously published in Eloe *et al.* [16].
(DOC)

Table S2 (A) Significantly different transporter classifications (TC IDs) identified between the deep and shallow metagenome comparisons. Highlighted in blue are the TC IDs that were differentially over-represented in the deep metagenomes. (B) Transporter family abundances for the five metagenomes.
(XLS)

Table S3 Detailed assembly and putative contaminant statistics for single-cell genomes. Mate pair ratio represents the total number of mated reads divided by the total number of reads. % Unique designates the percentage of reads after exclusion of duplicate reads, homopolymers, and removing N's. Clean datasets consisted of removal of all contigs less than 1 kb in length, as well as contigs greater than 1 kb with predicted proteins that had a phylogenetic affiliation different from the 16S rRNA phylogeny as determined using APIS.
(DOC)

Methods S1 **Preparation of DNA for 454 pyrosequencing.**
(DOC)
