# Supplementary figures and images for: Going Deeper: Metagenome of a Hadopelagic Microbial Community
Source: PLoS One. 2011 May 24;6(5):e20388. doi: 10.1371/journal.pone.0020388 (PMC3101246; doi:10.1371/journal.pone.0020388)

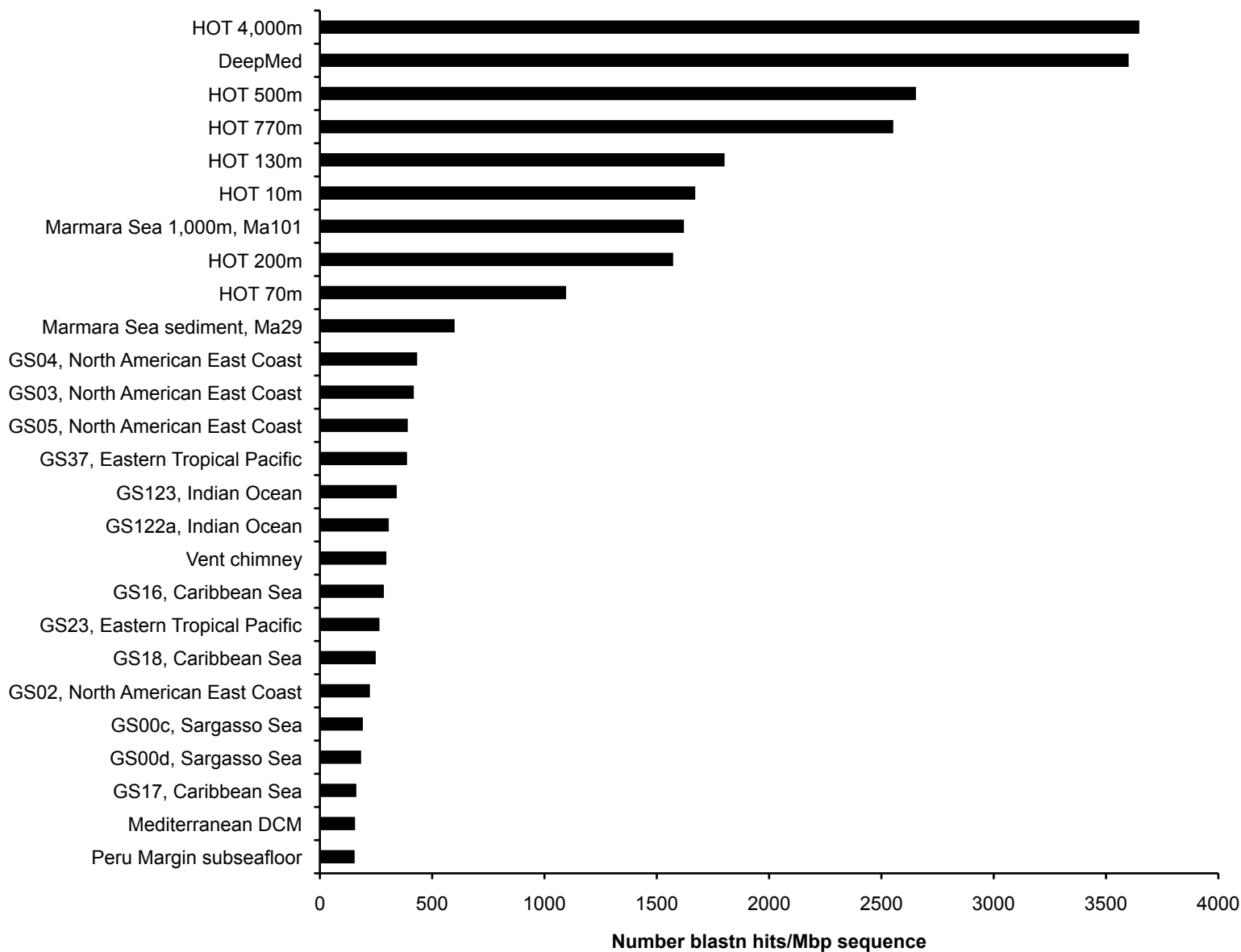

Supplement: Figure S1 — Comparison of the unassembled nonredundant PRT reads against the DeepMed [5]; a 7-depth profile from Station ALOHA (10 m, 70 m, 130 m, 200 m, 500 m, 770 m, 4,000 m) [7]; the Mediterranean deep chlorophyll maximum (DCM) [48]; 1,300 m depth sediment and 1,000 m depth water column from the Sea of Marmara [79]; black smoker chimney in the Mothra hydrothermal vent field at the Juan de Fuca Ridge [52]; the Peru Margin subseafloor [80]; and a subset of sites from the Sargasso Sea pilot study (GS00c and GS00d) [2] and the Global Ocean Survey (GS03, North American East Coast; GS04, North American East Coast; GS05, North American East Coast; GS16, Caribbean Sea; GS17, Caribbean Sea; GS18, Caribbean Sea; GS23, Eastern Tropical Pacific; GS37, Eastern Tropical Pacific; GS122a, Indian Ocean; GS123, Indian Ocean) [3], [4]. The number of top BLAST hits was normalized to the size of the comparison metagenome. (PDF) [file pone.0020388.s001.pdf]

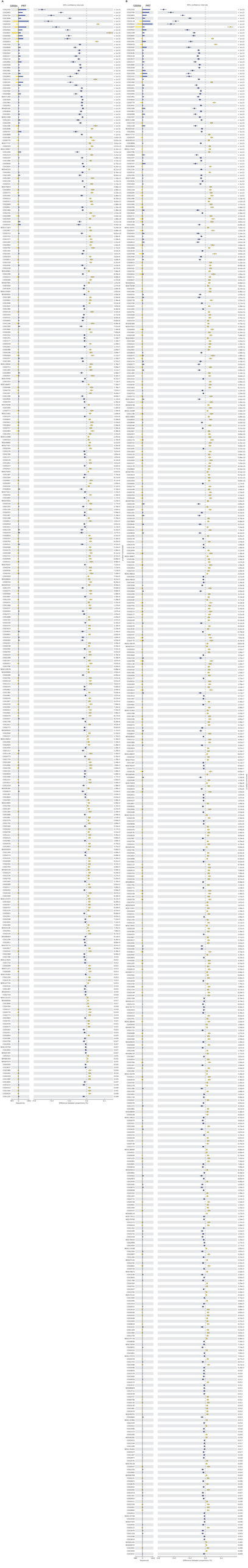

Supplement: Figure S2 — Statistical hypothesis testing implemented in the program STAMP [34] for differentially abundant orthologous groups (OGs) between the PRT metagenome and the Sargasso Sea (A) GS00c and (B) GS00d metagenomes. Results are shown for the Fisher's exact test using the Newcombe-Wilson method for calculating confidence intervals (CIs) at the 95% nominal coverage and a Bonferroni multiple test correction. Initially, 436 (PRT vs. GS00c) and 592 (PRT vs. GS00d) OGs were identified having significant differences (p<0.05). Subsequent filtering of these significant differences was performed taking into account effect size, with the difference between proportions set to a value of 0.5% and the ratio of proportions set to 2.0, resulting in 375 (PRT vs. GS00c) and 532 (PRT vs. GS00d) significantly different OGs represented. (PDF) [file pone.0020388.s002.pdf]

**A**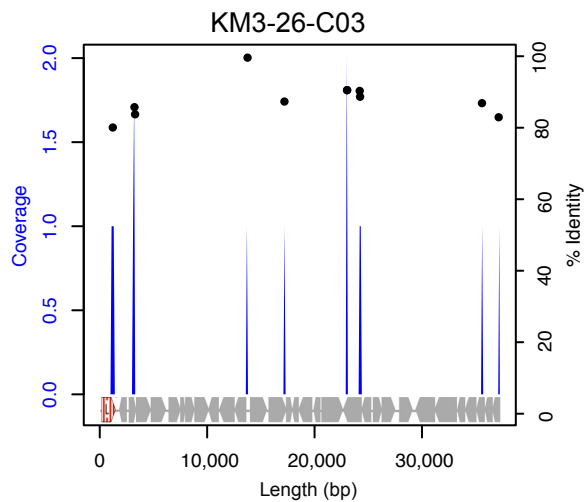**B**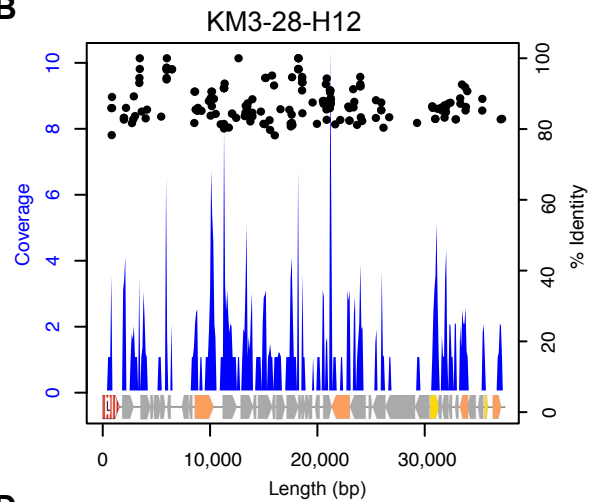**C**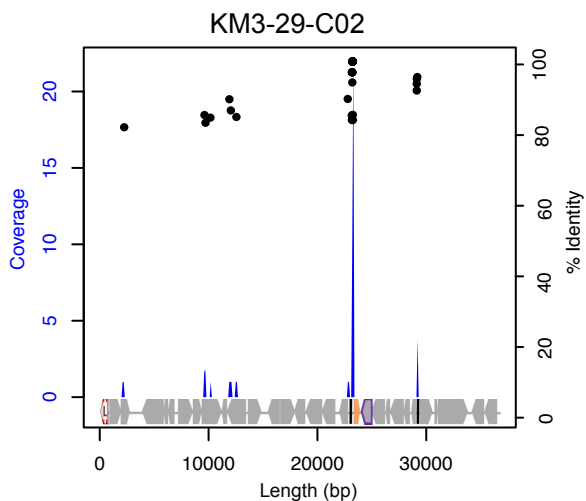**D**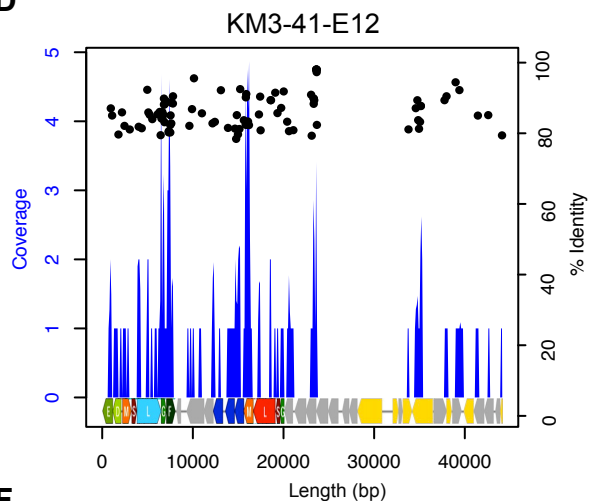**E**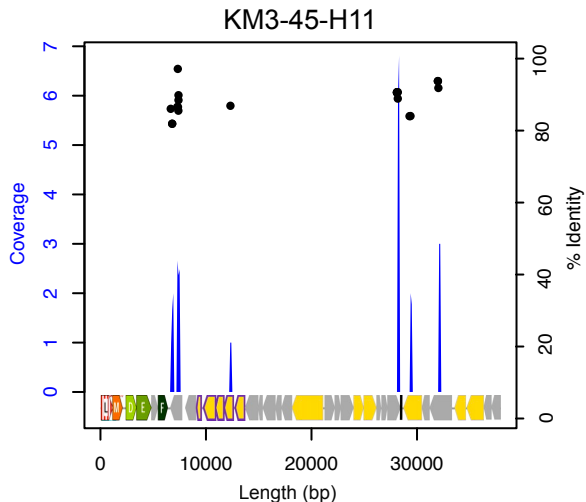**F**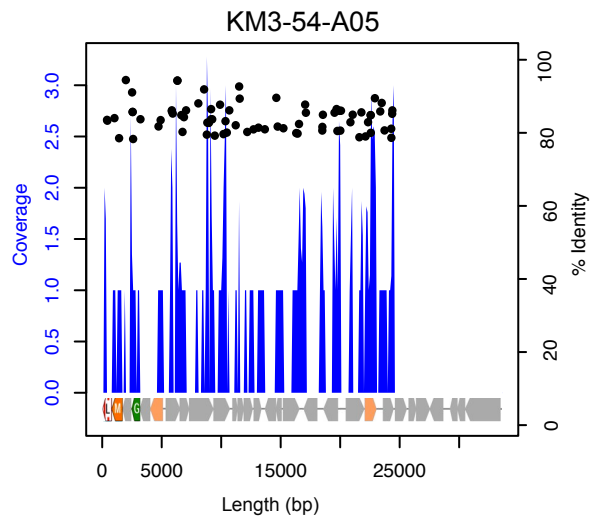**G**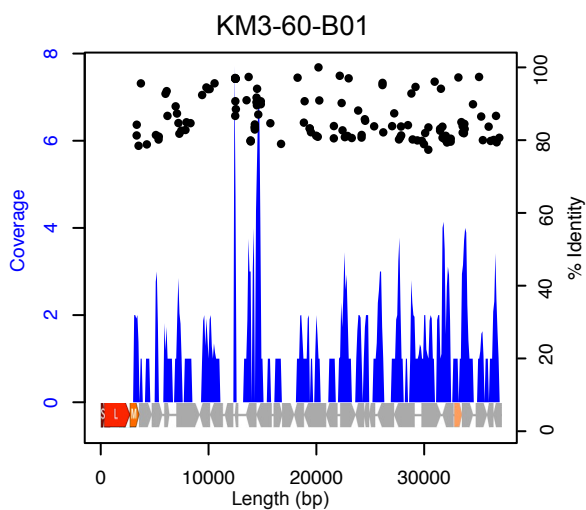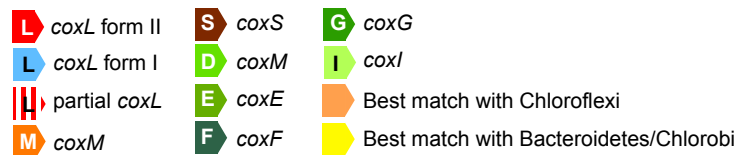

Supplement: Figure S3 — Fragment recruitment coverage plots for the unassembled nonredundant PRT metagenomic reads against the seven fully sequenced fosmids from Martín-Cuadrado et al. [49]. (A) KM3-26-C03 (NCBI Accession number: GU058051), (B) KM3-28-H12 (GU058052), (C) KM3-29-C02 (GU058053), (D) KM3-41-E12 (GU058054), (E) KM3-45-H11 (GU058057), (F) KM3-54-A05 (GU058055), (G) KM3-60-B01 (GU058056). Fragment recruitment was carried out using blastn as described by Rusch et al. [4]. Fosmid gene maps and annotations are shown as in Martín-Cuadrado et al. [49]. Coverage (blue bars) represents sequencing depth across the given fosmid, while % Identity (black circles) represents the percent sequence identity of the recruited PRT reads. (PDF) [file pone.0020388.s003.pdf]

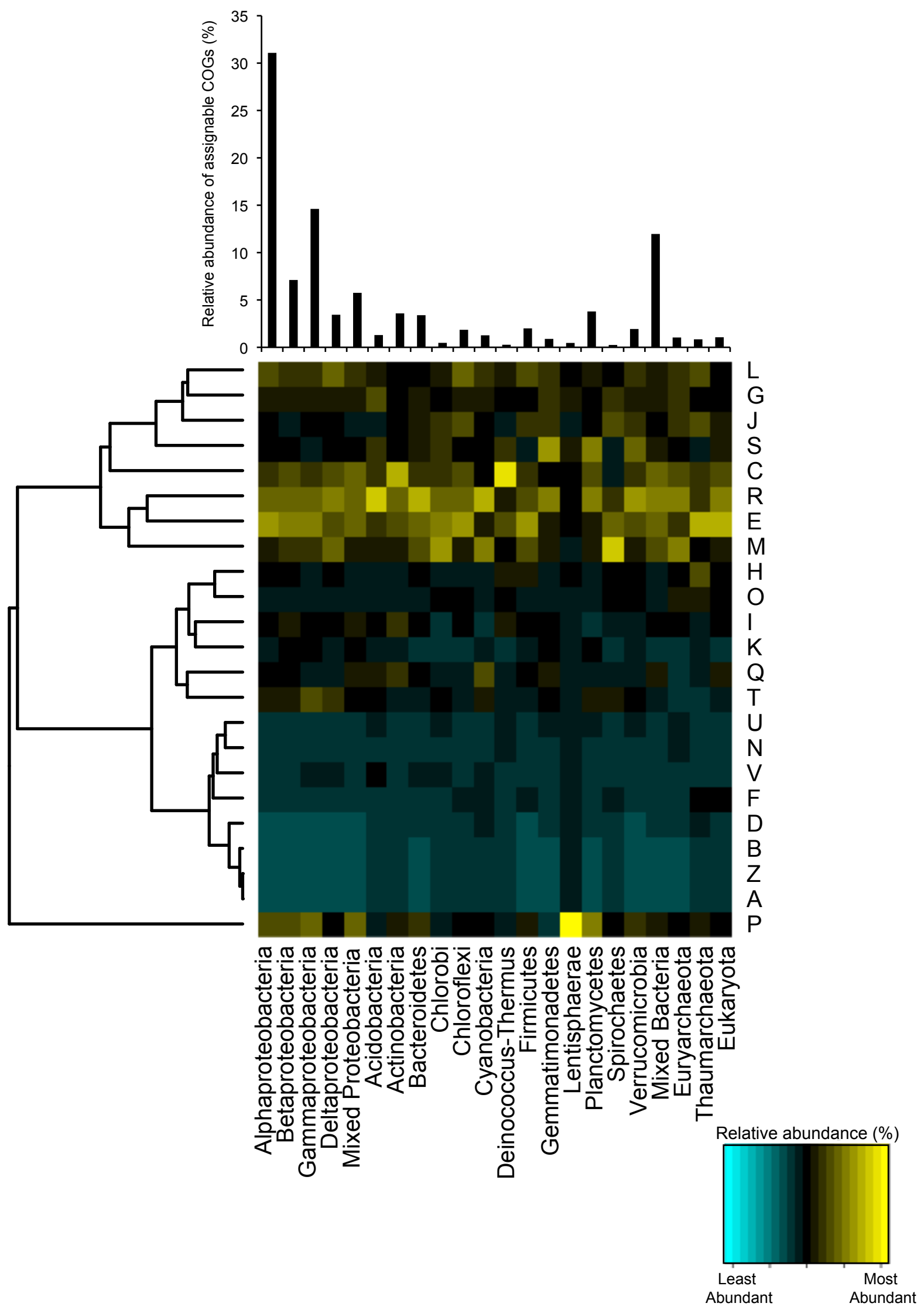

Supplement: Figure S7 — Relative abundance of assignable COG categories and distribution within phylum-level (and class-level for the Proteobacteria) groupings based on APIS. Only phyla contributing ≥0.2% of the total proteins classified are shown. COG categories are as follows: A, RNA processing and modification; B, chromatin structure and dynamics; C, energy production and conversion; D, cell division, chromosome partitioning; E, amino acid transport and metabolism; F, nucleotide transport and metabolism; G, carbohydrate transport and metabolism; H, coenzyme transport and metabolism; I, lipid transport and metabolism; J, translation and biogenesis; K, transcription; L, replication, recombination, and repair; M, cell wall/membrane/envelope; N, cell motility; O, protein turnover, chaperones; P, inorganic ion transport and metabolism; Q, secondary metabolism; R, general function prediction only; S, function unknown; T, signal transduction mechanisms; U, intracellular trafficking and secretion; V, defense mechanisms; and Z, cytoskeleton. (PDF) [file pone.0020388.s007.pdf]

**A**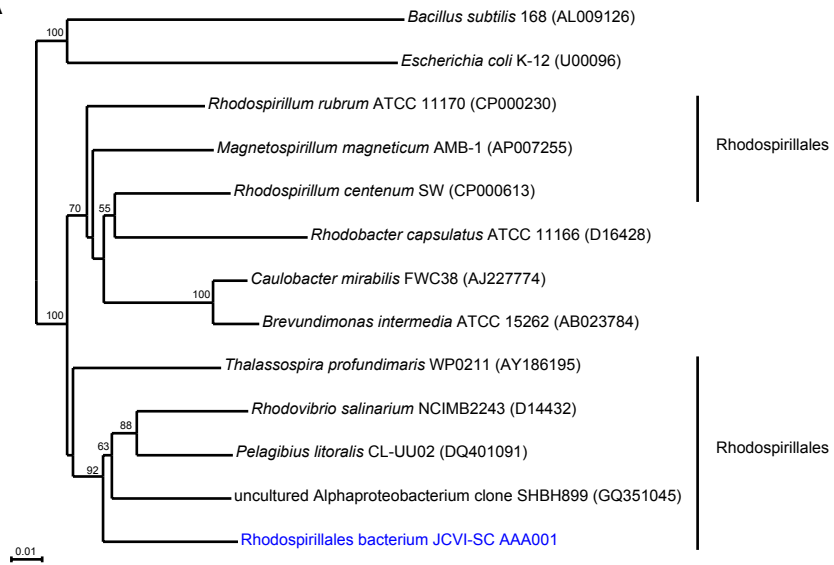**B**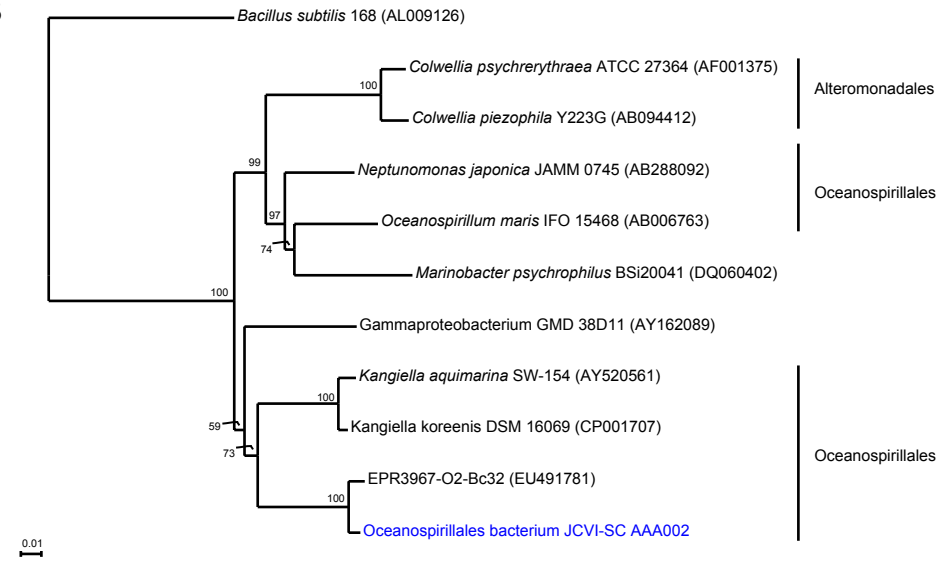**C**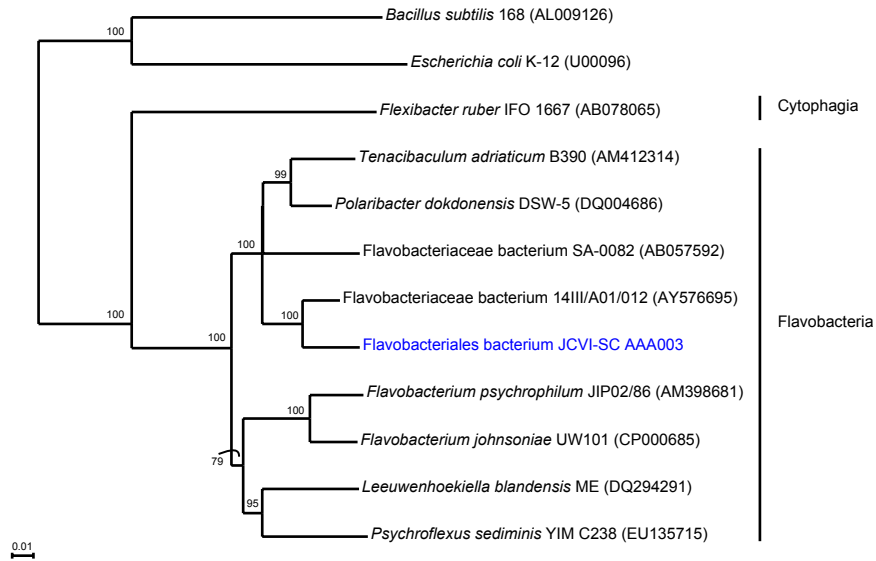**D**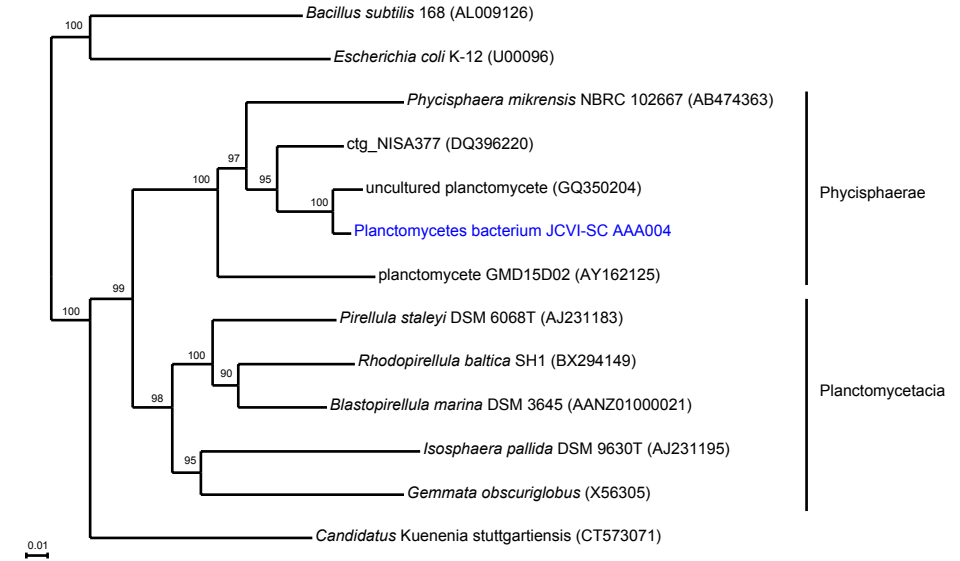

Supplement: Figure S8 — Phylogenetic trees depicting the relationship of the 16S rRNA gene sequences for the (A) Alphaproteobacterium Rhodospirillales bacterium JCVI-SC AAA001, (B) Gammaproteobacterium Oceanospirillales bacterium JCVI-SC AAA002, (C) Bacteroidetes Flavobacteriales bacterium JCVI-SC AAA003, and (D) Planctomycetes bacterium JCVI-SC AAA004. The rRNA gene sequences were aligned using the SINA Webaligner [23], uploaded into the ARB program [81] and manually checked with the ARB_EDIT4 tool. Aligned sequences were exported for bootstrap analysis using PHYLIP [82] for the neighbor joining method. Bootstrap support (1000 replicates) for nodes are indicated for values >50%. The outgroups used to calculate phylogeny were Bacillus subtilis 168 (AL009126) and Escherichia coli K-12 (U00096). (PDF) [file pone.0020388.s008.pdf]
