# Supplementary material for: Going Deeper: Metagenome of a Hadopelagic Microbial Community
Source: PLoS One. 2011 May 24;6(5):e20388. doi: 10.1371/journal.pone.0020388 (PMC3101246; doi:10.1371/journal.pone.0020388)
Supplement: Figure S4 — Abundance of the functional OG category Signal Transduction (T) for deep ocean metagenomes compared to the Sargasso Sea metagenomes. (PDF) [file pone.0020388.s004.pdf]

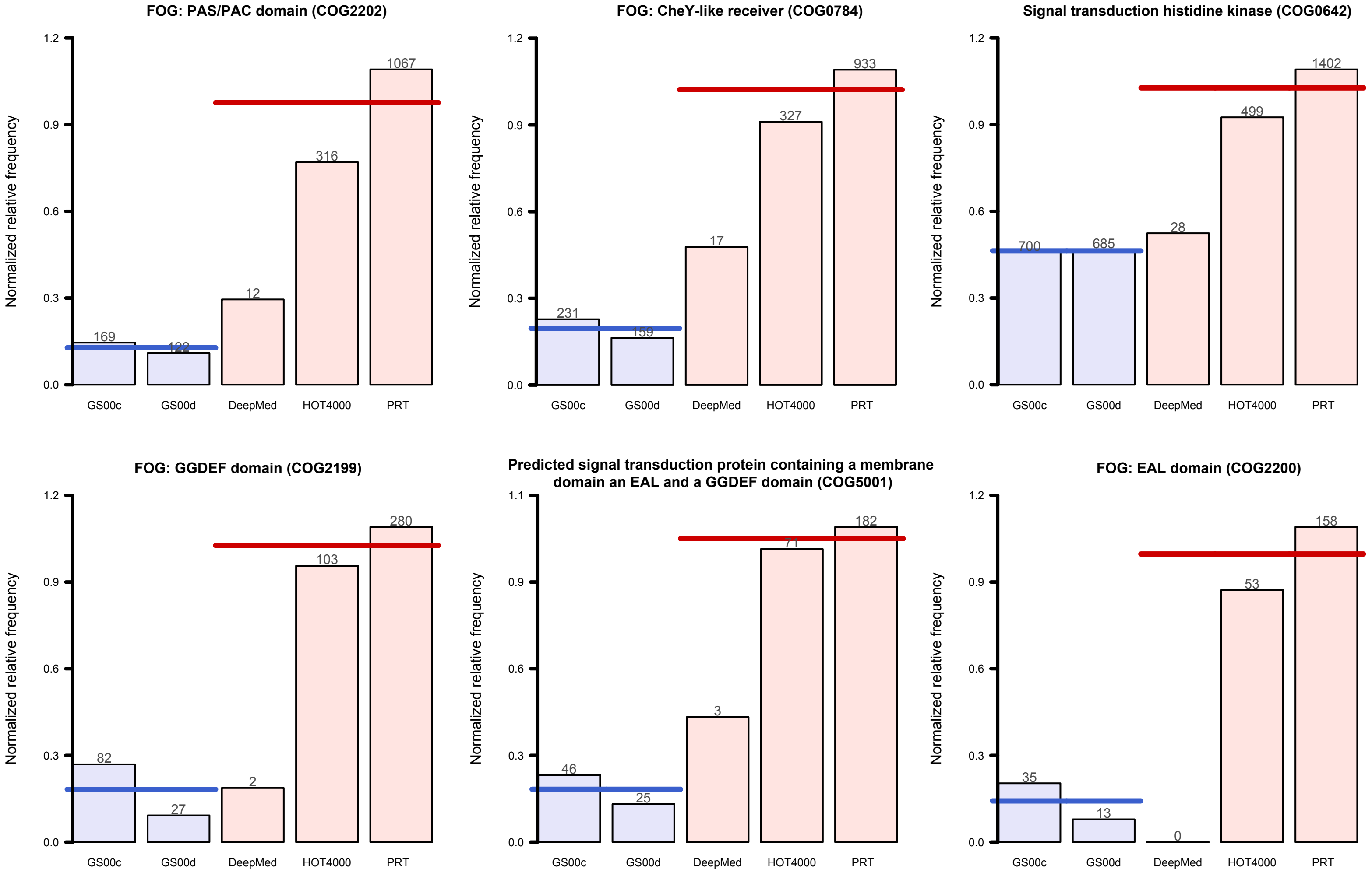

GeneFamily	Coefficient	AIC	P-value (BH)	Annotation	Class
COG2202	-2.03	102.81	6.41E-299	FOG: PAS/PAC domain	[T]
COG0784	-1.65	70.90	2.99E-214	FOG: CheY-like receiver	[T]
COG0642	-0.80	69.91	1.27E-110	Signal transduction histidine kinase	[T]
COG2199	-1.73	71.40	5.07E-67	FOG: GGDEF domain	[T]
COG5001	-1.75	39.92	9.37E-45	Predicted signal transduction protein containing a membrane domain an EAL and a GGDEF domain	[T]
COG2200	-1.95	50.33	1.11E-41	FOG: EAL domain	[T]
COG2203	-1.28	49.95	4.64E-40	FOG: GAF domain	[T]
COG2197	-1.92	44.04	4.28E-37	Response regulator containing a CheY-like receiver domain and an HTH DNA-binding domain	[TK]
COG3920	-2.43	33.44	3.23E-24	Signal transduction histidine kinase	[T]
COG2206	-1.72	35.94	2.48E-23	HD-GYP domain	[T]
COG4585	-2.24	24.82	2.88E-14	Signal transduction histidine kinase	[T]
COG3706	-1.27	44.41	1.16E-13	Response regulator containing a CheY-like receiver domain and a GGDEF domain	[T]
COG5278	-2.14	31.38	2.36E-13	Predicted periplasmic ligand-binding sensor domain	[T]
COG2766	-2.93	24.14	6.01E-13	Putative Ser protein kinase	[T]
COG1716	-2.13	25.90	5.89E-12	FOG: FHA domain	[T]
COG3300	-3.03	25.28	6.52E-11	MHYT domain (predicted integral membrane sensor domain)	[T]
COG4251	-2.48	28.36	1.03E-10	Bacteriophytochrome (light-regulated signal transduction histidine kinase)	[T]
COG0664	-0.74	37.00	1.44E-10	cAMP-binding proteins - catabolite gene activator and regulatory subunit of cAMP-dependent protein kinases	[T]
COG1639	-2.05	27.53	1.72E-07	Predicted signal transduction protein	[T]
COG4566	-3.07	21.11	2.07E-07	Response regulator	[T]
COG3614	-2.23	21.73	2.55E-07	Predicted periplasmic ligand-binding sensor domain	[T]
COG1217	-0.65	36.36	6.88E-07	Predicted membrane GTPase involved in stress response	[T]
COG2198	-1.64	25.18	4.25E-06	FOG: HPt domain	[T]
COG3605	-1.13	28.38	6.54E-06	Signal transduction protein containing GAF and PtsI domains	[T]
COG3322	-21.71	16.80	7.01E-05	Predicted periplasmic ligand-binding sensor domain	[T]
COG1966	-1.36	28.53	7.01E-05	Carbon starvation protein predicted membrane protein	[T]
COG2114	-0.32	38.11	7.53E-05	Adenylate cyclase family 3 (some proteins contain HAMP domain)	[T]
COG4191	-2.07	20.72	1.24E-04	Signal transduction histidine kinase regulating C4-dicarboxylate transport system	[T]
COG2204	-0.34	47.57	2.12E-04	Response regulator containing CheY-like receiver AAA-type ATPase and DNA-binding domains	[T]
COG2337	-2.66	18.55	3.51E-04	Growth inhibitor	[T]
COG2905	-2.66	18.46	3.51E-04	Predicted signal-transduction protein containing cAMP-binding and CBS domains	[T]
