# Supplementary material for: Going Deeper: Metagenome of a Hadopelagic Microbial Community
Source: PLoS One. 2011 May 24;6(5):e20388. doi: 10.1371/journal.pone.0020388 (PMC3101246; doi:10.1371/journal.pone.0020388)
Supplement: Figure S5 — Abundance of the functional OG category Transcription (K) for deep ocean metagenomes compared to the Sargasso Sea metagenomes. (PDF) [file pone.0020388.s005.pdf]

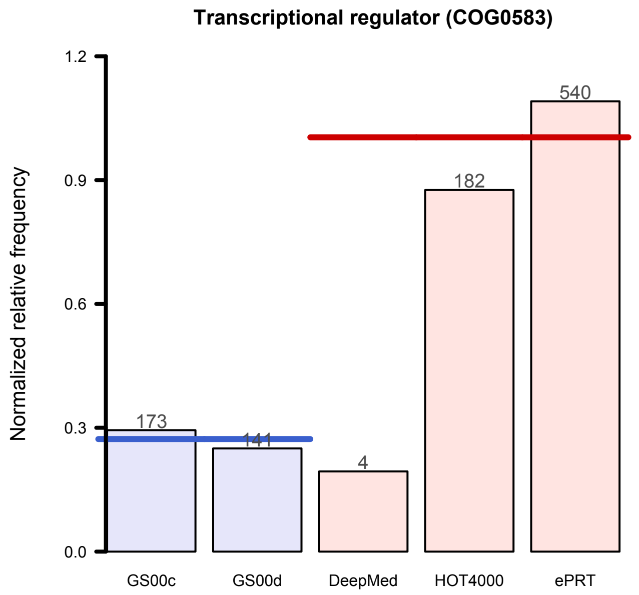

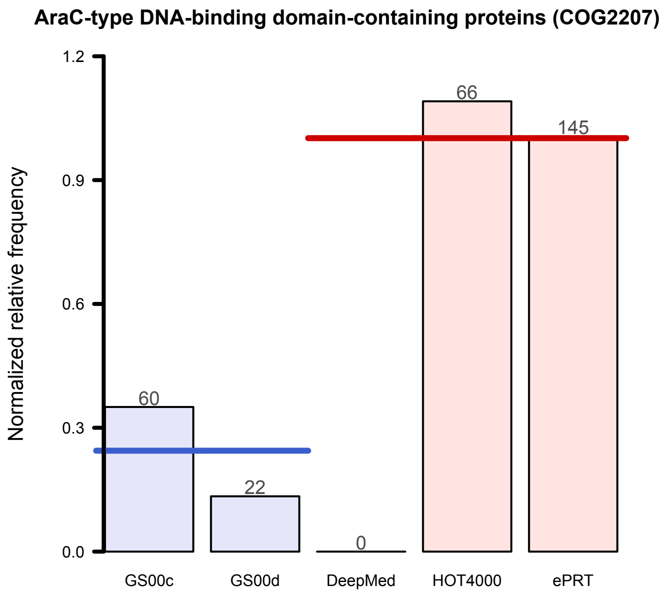

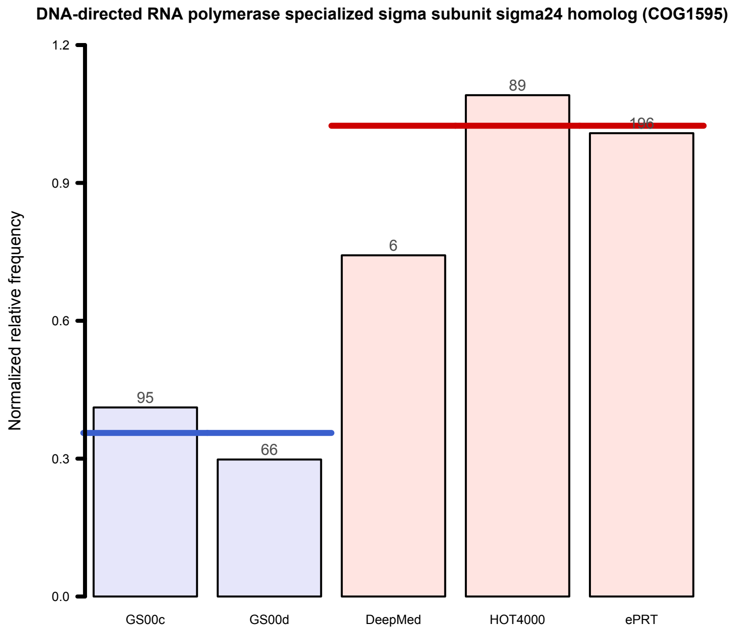

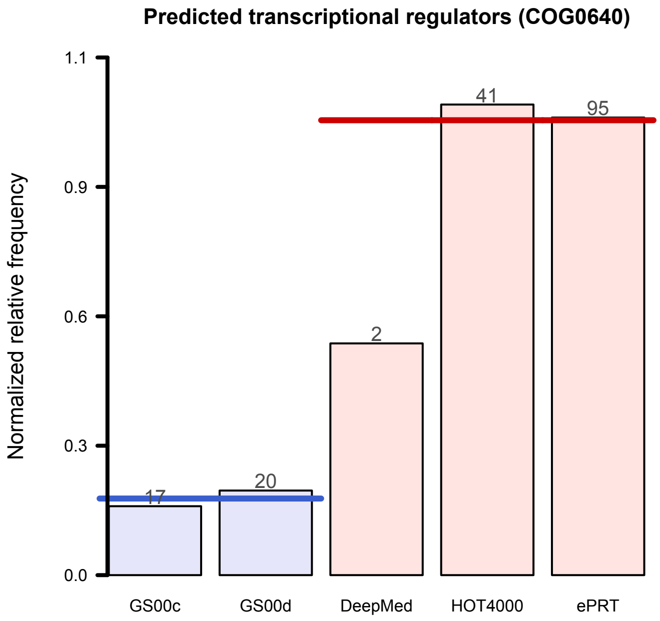

GeneFamily	Coefficient	AIC	P-value (BH)	Annotation	Class
COG0583	-1.30	65.62	7.64E-88	Transcriptional regulator	[K]
COG2207	-1.41	56.93	1.83E-27	AraC-type DNA-binding domain-containing proteins	[K]
COG1595	-1.06	38.72	6.19E-25	DNA-directed RNA polymerase specialized sigma subunit sigma24 homolog	[K]
COG0640	-1.78	29.68	4.70E-24	Predicted transcriptional regulators	[K]
COG1309	-0.94	49.31	5.21E-23	Transcriptional regulator	[K]
COG2771	-1.94	30.18	6.01E-15	DNA-binding HTH domain-containing proteins	[K]
COG1396	-0.85	42.21	4.86E-11	Predicted transcriptional regulators	[K]
COG3437	-1.69	25.79	4.51E-09	Response regulator containing a CheY-like receiver domain and an HD-GYP domain	[KT]
COG4977	-1.49	25.47	5.32E-08	Transcriptional regulator containing an amidase domain and an AraC-type DNA-binding HTH domain	[K]
COG2378	-1.83	24.02	5.36E-08	Predicted transcriptional regulator	[K]
COG2909	-2.73	22.51	2.71E-07	ATP-dependent transcriptional regulator	[K]
COG1522	-0.67	40.03	4.70E-07	Transcriptional regulators	[K]
COG1733	-1.54	24.81	8.22E-07	Predicted transcriptional regulators	[K]
COG1802	-0.88	32.83	1.63E-06	Transcriptional regulators	[K]
COG2186	-1.31	25.15	4.63E-06	Transcriptional regulators	[K]
COG1695	-1.61	24.55	8.17E-06	Predicted transcriptional regulators	[K]
COG4941	-3.46	20.47	8.66E-06	Predicted RNA polymerase sigma factor containing a TPR repeat domain	[K]
COG2808	-2.54	19.60	1.55E-05	Transcriptional regulator	[K]
COG1349	-1.85	28.08	2.60E-05	Transcriptional regulators of sugar metabolism	[KG]
COG4957	-2.50	21.08	3.43E-05	Predicted transcriptional regulator	[K]
COG3829	-3.30	17.17	1.14E-04	Transcriptional regulator containing PAS AAA-type ATPase and DNA-binding domains	[KT]
COG2183	-1.35	29.49	1.92E-04	Transcriptional accessory protein	[K]
COG3604	-1.44	24.56	2.23E-04	Transcriptional regulator containing GAF AAA-type ATPase and DNA binding domains	[KT]
COG4646	-2.66	19.80	3.51E-04	DNA methylase	[KL]
