# Supplementary material for: Going Deeper: Metagenome of a Hadopelagic Microbial Community
Source: PLoS One. 2011 May 24;6(5):e20388. doi: 10.1371/journal.pone.0020388 (PMC3101246; doi:10.1371/journal.pone.0020388)
Supplement: Figure S6 — Abundance of the functional OG category Inorganic ion transport and metabolism (P) for deep ocean metagenomes compared to the Sargasso Sea metagenomes. (PDF) [file pone.0020388.s006.pdf]

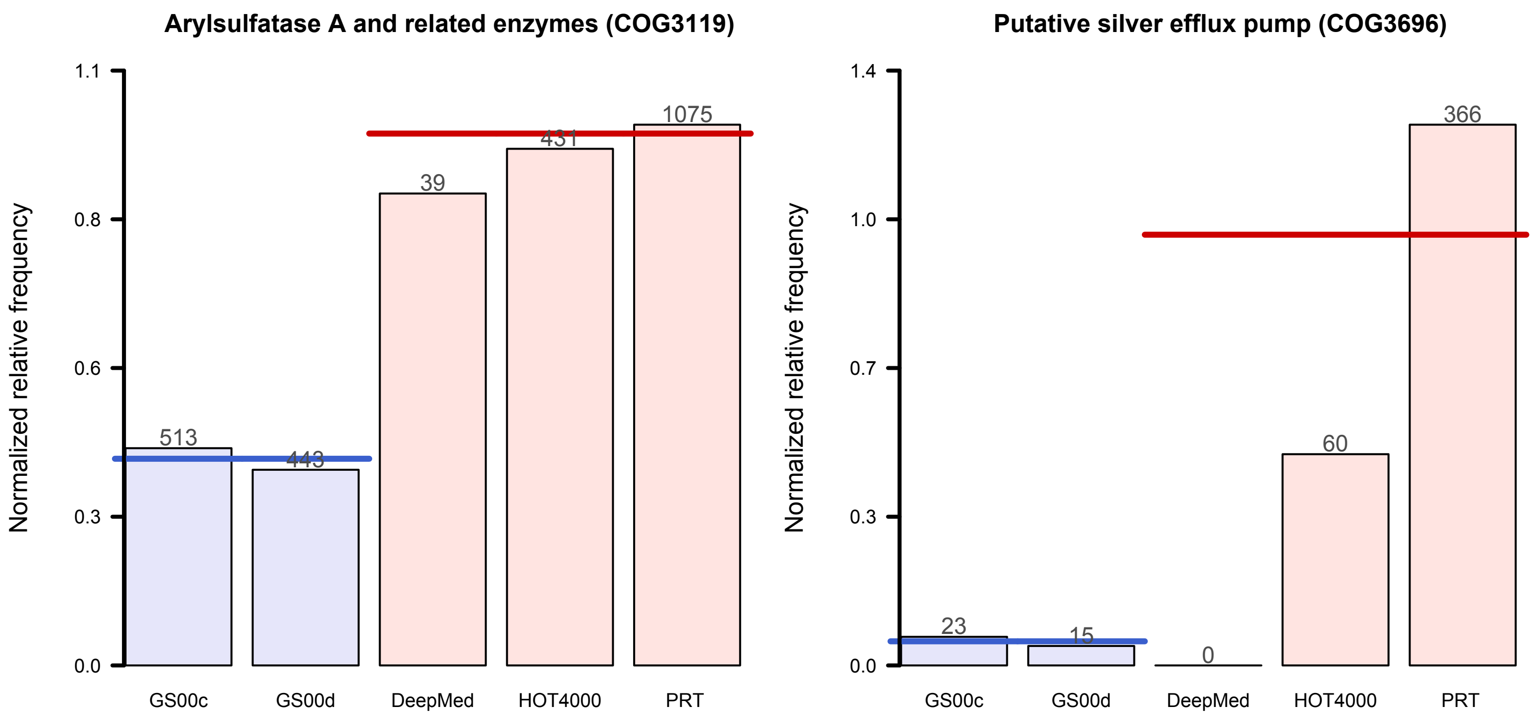

GeneFamily	Coefficient	AIC	P-value (BH)	Annotation	Class
COG3696	-2.88	109.60	1.25E-124	Putative silver efflux pump	[P]
COG3119	-0.95	46.09	1.46E-116	Arylsulfatase A and related enzymes	[P]
COG2217	-1.43	53.54	2.38E-50	Cation transport ATPase	[P]
COG1230	-1.93	37.86	3.77E-24	Co/Zn/Cd efflux system component	[P]
COG0155	-1.38	31.38	6.33E-17	Sulfite reductase beta subunit (hemoprotein)	[P]
COG0715	-1.09	35.41	2.59E-16	ABC-type nitrate/sulfonate/bicarbonate transport systems periplasmic components	[P]
COG0753	-3.10	28.47	3.13E-16	Catalase	[P]
COG3158	-21.81	27.95	5.54E-16	K+ transporter	[P]
COG3667	-2.84	22.07	1.70E-11	Uncharacterized protein involved in copper resistance	[P]
COG1629	-0.25	221.37	5.47E-11	Outer membrane receptor proteins mostly Fe transport	[P]
COG4773	-2.39	32.78	2.44E-09	Outer membrane receptor for ferric coprogen and ferric-rhodotorulic acid	[P]
COG0370	-1.56	29.81	6.47E-09	Fe2+ transport system protein B	[P]
COG0659	-0.91	35.65	1.24E-08	Sulfate permease and related transporters (MFS superfamily)	[P]
COG4774	-2.80	26.09	5.26E-08	Outer membrane receptor for monomeric catechols	[P]
COG0475	-0.91	35.23	1.82E-07	Kef-type K+ transport systems membrane components	[P]
COG0053	-1.23	29.24	2.51E-07	Predicted Co/Zn/Cd cation transporters	[P]
COG0598	-2.23	25.24	2.55E-07	Mg2+ and Co2+ transporters	[P]
COG2060	-22.91	13.74	4.53E-06	K+-transporting ATPase A chain	[P]
COG3712	-1.70	26.79	9.11E-06	Fe2+-dicitrate sensor membrane component	[PT]
COG0025	-0.96	43.81	9.98E-06	NhaP-type Na+/H+ and K+/H+ antiporters	[P]
COG3131	-3.41	16.55	2.05E-05	Periplasmic glucans biosynthesis protein	[P]
COG1116	-0.77	30.68	5.57E-05	ABC-type nitrate/sulfonate/bicarbonate transport system ATPase component	[P]
COG0306	-0.82	30.19	7.62E-05	Phosphate/sulphate permeases	[P]
COG2216	-21.64	14.88	1.70E-04	High-affinity K+ transport system ATPase chain B	[P]
COG3746	-1.63	23.88	1.83E-04	Phosphate-selective porin	[P]
COG2223	-1.63	22.11	1.83E-04	Nitrate/nitrite transporter	[P]
COG4638	-0.32	41.92	2.16E-04	Phenylpropionate dioxygenase and related ring-hydroxylating dioxygenases large terminal subunit	[PR]
COG0600	-0.62	44.97	4.78E-04	ABC-type nitrate/sulfonate/bicarbonate transport system permease component	[P]
