# Supplementary material for: Going Deeper: Metagenome of a Hadopelagic Microbial Community
Source: PLoS One. 2011 May 24;6(5):e20388. doi: 10.1371/journal.pone.0020388 (PMC3101246; doi:10.1371/journal.pone.0020388)
Supplement: Table S1 — Chemical and biological constituents of hadal (6,000 m) seawater. Data previously published in Eloe et al. [16]. (DOC) [file pone.0020388.s009.doc]

**Table S1.** Chemical and biological constituents of hadal (6,000 m) seawater. Data previously published in Eloe *et al.* [16].

|  | PRT (6,000 m) |
| --- | --- |
| N+N (µmol l^-1^) | 24.18 ± 0.70 |
| PO_4_ (µmol l^-1^) | 1.67 ± 0.01 |
| Silicate (µmol l^-1^) | 63.77 ± 0.25 |
| NO_2_ (µmol l^-1^) | 0 |
| NH_4_ (µmol l^-1^) | 0.043 ± 0.0058 |
| NO_3_ (µmol l^-1^) | 24.18 ± 0.70 |
| O_2_ (µmol kg^-1^) | 240 |
| Salinity | 34.84 |
| TON (µM) | 29.3 ± 0.76 |
| TOC (µM) | 46.2 ± 2.06 |
| Viral abundance (particles ml^-1^) | 4.50 x 10^5^ |
| Microbial abundance (cells ml^-1^) | 1.09 x 10^4^ |
