# Supplementary material for: Going Deeper: Metagenome of a Hadopelagic Microbial Community
Source: PLoS One. 2011 May 24;6(5):e20388. doi: 10.1371/journal.pone.0020388 (PMC3101246; doi:10.1371/journal.pone.0020388)
Supplement: Table S3 — Detailed assembly and putative contaminant statistics for single-cell genomes. Mate pair ratio represents the total number of mated reads divided by the total number of reads. % Unique designates the percentage of reads after exclusion of duplicate reads, homopolymers, and removing N's. Clean datasets consisted of removal of all contigs less than 1 kb in length, as well as contigs greater than 1 kb with predicted proteins that had a phylogenetic affiliation different from the 16S rRNA phylogeny as determined using APIS. (DOC) [file pone.0020388.s011.doc]

**Table S3.** Detailed assembly and putative contaminant statistics for single-cell genomes. Mate pair ratio represents the total number of mated reads divided by the total number of reads. % Unique designates the percentage of reads after exclusion of duplicate reads, homopolymers, and removing N’s. Clean datasets consisted of removal of all contigs less than 1 kb in length, as well as contigs greater than 1 kb with predicted proteins that had a phylogenetic affiliation different from the 16S rRNA phylogeny as determined using APIS.

|  | Alphaproteobacterium Rhodospirillales bacterium JCVI-SC AAA001 | Bacteroidetes Flavobacteriales bacterium JCVI-SC AAA003 | Gammaproteobacterium Oceanospirillales bacterium JCVI-SC AAA002 | Planctomycetes bacterium JCVI-SC AAA004 |
| --- | --- | --- | --- | --- |
| Total # reads used for assembly | 113,421 | 130,439 | 189,378 | 42,704 |
| Total bases used for assembly (bp) | 24,465,204 | 27,133,706 | 37,000,539 | 11,443,900 |
| Mate Pair ratio | 70% | 64% | 80% | 4% |
| % Unique | 80.06% | 75.32% | 79.91% | 85.07% |
| Q20+ % | 99.90% | 99.82% | 99.88% | 99.91% |
| Total assembled contigs (bp) | 471,029 | 241,120 | 282,158 | 159,263 |
| Total # contigs | 249 | 84 | 66 | 144 |
| # contigs  < 1kb | 188 | 70 | 44 | 114 |
| Putative contaminant sequences > 1kbp (bp) | 60,520 | 0 | 69,190 | 38,950 |
| Clean assembled contigs (bp) | 310,294 | 209,062 | 190,170 | 58,361 |
