# Supplementary material for: Going Deeper: Metagenome of a Hadopelagic Microbial Community
Source: PLoS One. 2011 May 24;6(5):e20388. doi: 10.1371/journal.pone.0020388 (PMC3101246; doi:10.1371/journal.pone.0020388)
Supplement: Methods S1 — Preparation of DNA for 454 pyrosequencing. (DOC) [file pone.0020388.s012.doc]

**SUPPLEMENTARY METHODS**

**Preparation of DNA for 454 pyrosequencing.** DNA was randomly sheared, end polished with Bal31 nuclease/T4 DNA Polymerase (NE Biolabs), and carefully size-selected on a 1% low-melting-point agarose gel. Adaptors were appended to the DNA fragments followed by amplification with Phusion DNA Polymerase (Finnzymes, Espoo, Finland) for 15 cycles. Amplified DNA fragments were purified through three rounds of gel purification using 1% low-melting-point agarose (Invitrogen). DNA was fragmented further and 500 – 800bp fragments were size-selected. Adaptors, serving as priming sites for amplification and sequencing, were ligated onto the fragments. After library quantification, amplification was carried out in an emulsion-based format and subsequently purified using AMPure beads (Beckman Coulter), selectively binding targeted DNA fragments while allowing for the removal of primers and other small DNA fragments. The library was processed through the breaking and enriching steps, followed by sequencing on the Genome Sequencer FLX System (454 Life Sciences) at the Joint Technology Center of the J. Craig Venter Institute (Rockville, MD).

**454 Pyrosequence Processing.** A half plate of sequence data was produced, resulting in 620,565 reads (average read length, 390 bp). A default Newbler assembly yielded 25,776 assembled contigs (number assembled reads, 180,036 [29.01%]). The singleton data consisted of a total of 440,782 reads. After the 454 replicate filter, 331,384 reads were found to be unique with 24.82% of the singleton dataset marked as artificial replicates based on the 90% sequence identity threshold. The 331,384 nonredundant, singleton reads had an average read length of 392 bp (minimum, 11 bp; maximum, 667 bp).

**Functional annotations.** All nonredundant proteins were searched using blastp against the STRING database (v8.3) [1] to assign top hits to orthologous groups (OGs) in the eggNOG database, as well as queried against the KEGG orthologs (KOs) and Pfam database. E-value cut-offs for all blastp queries were set at e^-5^. For OGs and Pfam searches, top hits were allowed to map to multiple OG categories and multiple Pfam motifs per query. The nonredundant proteins were additionally queried using blastp as described above against the Transporter Classification Database to assign transporter classifications (TC IDs) for membrane transport proteins [2].

**Metagenome comparisons.** The unassembled nonredundant singleton reads were searched using blastn with an E-value cutoff of e^-5^ against the following available marine metagenomes: the DeepMed [3]; a 7-depth profile from Station ALOHA (10 m, 70 m, 130 m, 200 m, 500 m, 770 m, 4,000 m) [4]; the Mediterranean deep chlorophyll maximum (DCM) [5]; 1,300 m depth sediment and 1,000 m depth water column from the Sea of Marmara [6]; black smoker chimney in the Mothra hydrothermal vent field at the Juan de Fuca Ridge [7]; the Peru Margin subseafloor [8]; and a subset of sites from the Sargasso Sea pilot study (GS00c and GS00d) [9] and the Global Ocean Survey (GS03, North American East Coast; GS04, North American East Coast; GS05, North American East Coast; GS16, Caribbean Sea; GS17, Caribbean Sea; GS18, Caribbean Sea; GS23, Eastern Tropical Pacific; GS37, Eastern Tropical Pacific; GS122a, Indian Ocean; GS123, Indian Ocean) [10,11] (Fig. S1).

For quantitative metagenome comparisons, the nonredundant functionally annotated protein datasets from the PRT, HOT4000, DeepMed, GS00c, and GS00d were used. Pair-wise direct comparisons were carried out in the program ShotgunFunctionalizeR [12] using a binomial method with the Benjamini-Hochberg False Discovery Rate correction to adjust q-values for multiple testing. Read length was not taken into account in these comparisons since annotated proteins were compared from the five datasets, however the metagenome sizes and gene stochiometries were normalized using a generalized linear model with the Poisson canonical logarithmic link function. The total number of reads assigned to a particular orthologous group, Pfam, or KO for each metagenome was used as an offset to remove the effects of unequal sample sizes as implemented in the program ShotgunFunctionalizeR [12]. Additionally, direct comparisons between the groups “shallow” (referring to surface seawater derived datasets, GS00c and GS00d) versus “deep” (referring to the three datasets derived from depth: DeepMed, HOT4000, and PRT) enabled testing for gene families that were significantly represented in deep versus shallow metagenomes using the Poisson model with normalization. A coefficient from the Poisson model, which is the estimated difference between the two groups, as well as the Akaike information criterion (AIC) as a measure of the model fit, are reported for all of the OG families tested (Figs. S4-6).

Additionally, a more rigorous pairwise comparison for the PRT nonredundant proteins and the two Sargasso Sea datasets was performed using the statistical techniques available within the program STAMP. This method allowed functional profile comparisons taking into account effect size and the difference between proportions [13]. The statistical hypothesis test implemented was Fisher’s exact test using the Newcombe-Wilson method for calculating confidence intervals (CIs) at the 95% nominal coverage and a Bonferroni multiple test correction.

A short discussion is warranted here for these differential comparisons with regard to genome size differences estimated from the surface- and deep-ocean metagenomes. Considering the average EGS for the PRT metagenome compared to the two Sargasso Sea metagenomes is approximately 2-fold greater, the calculated differences in the functional gene abundance profiles is in part a reflection of this increase in genome size [14]. However, considering the estimate uncertainty associated with incorporating this biasing factor is at this time unclear, we have refrained from including the EGS in our quantitative comparative analyses to avoid unwanted propagation of this uncertainty.

**Assembly of 454-pyrosequencing MDA data.** MDA samples present challenges during assembly of the sequencing data, which include both over- and under-representation of regions of the genome, chimera formation, and sometimes the presence of contaminating DNAs [15,16]. In the analysis of the four deep trench samples, we addressed these issues in the following manner. Reads and contigs from initial *de novo* Newbler assembly were processed through the JCVI metagenomic 16S rRNA pipeline and searched against the PANDA database to provide taxonomic classification of ribosomal and non-ribosomal sequences [17]. Because over amplification of random regions creates uneven distribution of coverage, the contigs from the initial *de novo* assembly were screened for short contigs with high coverage. The reads from these tagged contigs were reduced in coverage and the filtered dataset was reassembled. The Newbler read type classification was used from a reference mapping assembly to screen for chimeric reads. The initial *de novo* consensus was used as a reference in a mapping assembly of the raw reads. Potential chimeric reads were removed from the readset and the filtered dataset was reassembled. This final iteration of assembly resulted in the non-chimeric assembly from which orf calling and functional annotation was performed.

Assemblies were manually curated using a conservative approach as follows. All contigs less than 1,000 bp in length were removed. Taxonomic affiliations of the predicted protein sequences assigned using APIS [18], and contigs which contained a majority of proteins with taxonomic affiliations other than the phylum-level classification associated with the 16S rRNA gene were removed. While this curation removed a sizeable fraction of the single cell sequence data downstream analysis (Supplementary Table S3), it rendered confidence in the final datasets since taxonomic affiliation of the proteins was in accordance with the 16S rRNA phylogeny.

**References**

1. Jensen LJ, Kuhn M, Stark M, Chaffron S, Creevey C, et al. (2009) STRING 8-a global view on proteins and their functional interactions in 630 organisms. Nucleic Acids Res 37: D412-D416.

2. Saier MH, Yen MR, Noto K, Tamang DG, Elkan C (2009) The Transporter Classification Database: recent advances. Nucleic Acids Res 37: D274-D278.

3. Martín-Cuadrado AB, López-García P, Alba JC, Moreira D, Monticelli L, et al. (2007) Metagenomics of the Deep Mediterranean, a Warm Bathypelagic Habitat. PLoS One 2: e914.

4. DeLong EF, Preston CM, Mincer T, Rich V, Hallam SJ, et al. (2006) Community genomics among stratified microbial assemblages in the ocean's interior. Science 311: 496-503.

5. Ghai R, Martín-Cuadrado AB, Molto AG, Heredia IG, Cabrera R, et al. (2010) Metagenome of the Mediterranean deep chlorophyll maximum studied by direct and fosmid library 454 pyrosequencing. ISME J 4: 1154-1166.

6. Quaiser A, Zivanovic Y, Moreira D, López-García P (2011) Comparative metagenomics of bathypelagic plankton and bottom sediment from the Sea of Marmara. ISME J 5: 285-304.

7. Xie W, Wang F, Guo L, Chen Z, Sievert SM, et al. (2011) Comparative metagenomics of microbial communities inhabiting deep-sea hydrothermal vent chimneys with contrasting chemistries. ISME J 5: 414-426.

8. Biddle JF, Fitz-Gibbon S, Schuster SC, Brenchley JE, House CH (2008) Metagenomic signatures of the Peru Margin subseafloor biosphere show a genetically distinct environment. Proc Natl Acad Sci U S A 105: 10583-10588.

9. Venter JC, Remington K, Heidelberg JF, Halpern AL, Rusch D, et al. (2004) Environmental genome shotgun sequencing of the Sargasso Sea. Science 304: 66-74.

10. Yooseph S, Nealson KH, Rusch DB, McCrow JP, Dupont CL, et al. (2010) Genomic and functional adaptation in surface ocean planktonic prokaryotes. Nature 468: 60-66.

11. Rusch DB, Halpern AL, Sutton G, Heidelberg KB, Williamson S, et al. (2007) The Sorcerer II Global Ocean Sampling expedition: Northwest Atlantic through Eastern Tropical Pacific. PLoS Biol 5: 398-431.

12. Kristiansson E, Hugenholtz P, Dalevi D (2009) ShotgunFunctionalizeR: an R-package for functional comparison of metagenomes. Bioinformatics 25: 2737-2738.

13. Parks DH, Beiko RG (2010) Identifying biologically relevant differences between metagenomic communities. Bioinformatics 26: 715-721.

14. Beszteri B, Temperton B, Frickenhaus S, Giovannoni SJ (2010) Average genome size: a potential source of bias in comparative metagenomics. ISME J 4: 1075-1077.

15. Rodrigue S, Malmstrom RR, Berlin AM, Birren BW, Henn MR, et al. (2009) Whole genome amplification and *de novo* assembly of single bacterial cells. PLoS One 4: e6864.

16. Raghunathan A, Ferguson HR, Jr., Bornarth CJ, Song W, Driscoll M, et al. (2005) Genomic DNA Amplification from a Single Bacterium. Appl Environ Microbiol 71: 3342-3347.

17. Tanenbaum DM, Goll J, Murphy S, Kumar P, Zafar N, et al. (2010) The JCVI standard operating procedure for annotating prokaryotic metagenomic shotgun sequencing data. Stand Genomic Sci 2: 229.

18. Badger JH, Hoover TR, Brun YV, Weiner RM, Laub MT, et al. (2006) Comparative genomic evidence for a close relationship between the dimorphic prosthecate bacteria *Hyphomonas neptunium* and *Caulobacter crescentus*. J Bacteriol 188: 6841-6850.
